# Supplementary figures and images for: Integration, coincidence detection and resonance in networks of spiking neurons expressing Gamma oscillations and asynchronous states
Source: PLoS Comput Biol. 2021 Sep 16;17(9):e1009416. doi: 10.1371/journal.pcbi.1009416 (PMC8478196; doi:10.1371/journal.pcbi.1009416)

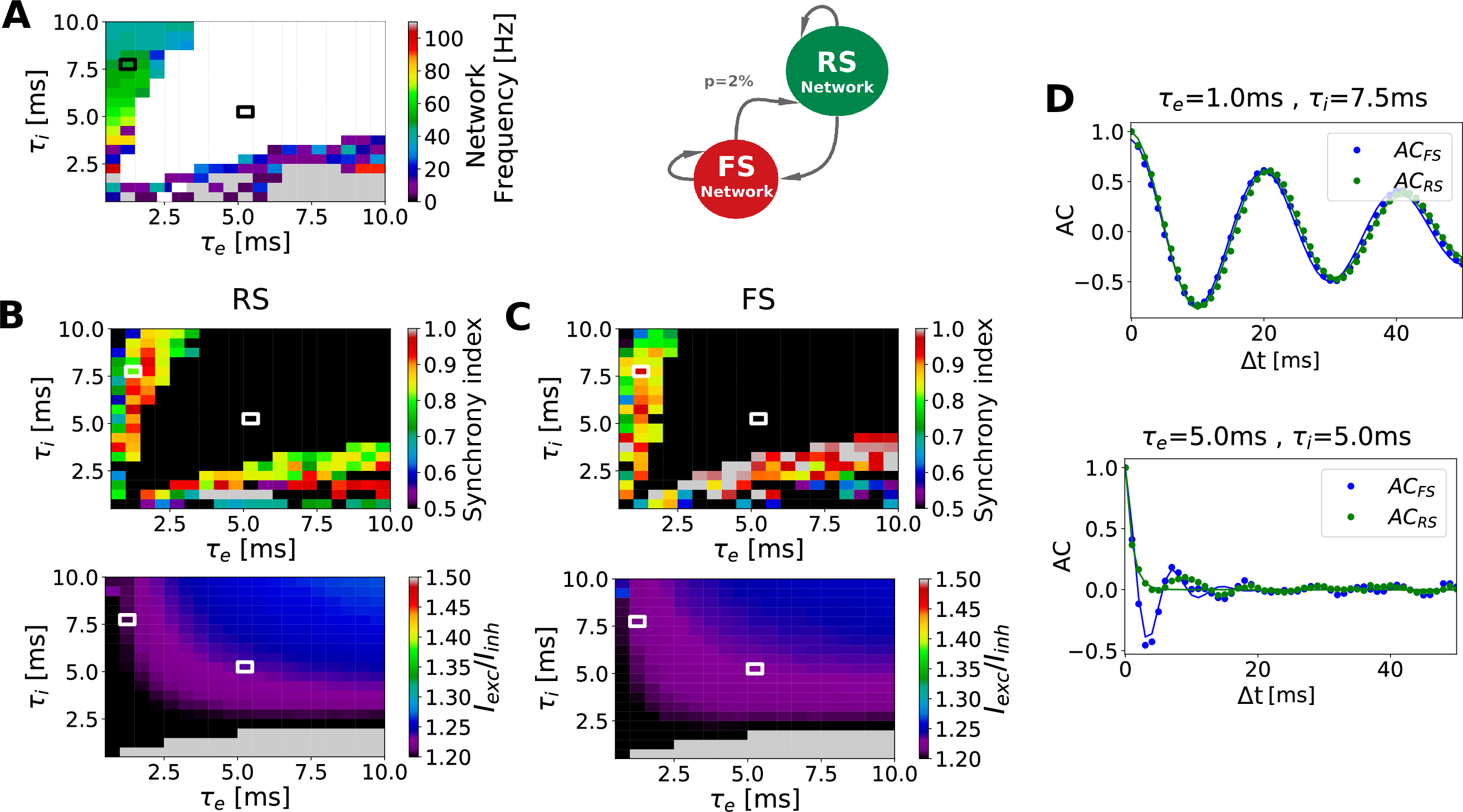

Supplement: S1 Fig — The network used to produce this figure was composed of 20000 excitatory Regular Spiking and 5000 inhibitory Fast Spiking neurons connected randomly with a probability of connection of 2%. All synapses were delayed by a time delay of 1.5 ms, and had reference synaptic strengths of QeR=1 nS or QiR=5 nS and reference synaptic time scales of τeR=τiR=5 ms. Synaptic strengths (Qe,i) were normalized at each tested time scale (τe,i) to keep the same synaptic gain, such that: Qe,i=(Qe,iR.τe,iR)/τe,i. A: Network oscillation frequency depicted in a color scheme as a function of excitatory and inhibitory synaptic time scales. White color corresponds to regions in which no oscillation was identified in RS population. B: Synchrony Index of RS population (top) and network balance (bottom) as a function of synaptic time scales. The Synchrony Index (SI) is based on the auto-correlation of the population frequency of RS cells. To be calculated, the autocorrelation of the population frequency was fitted by a damped cosine function and the value of this fitted function at zero time lag was defined as the SI. If the exponential decay rate was higher then 100, it was considered that there was no global oscillation at the population scale. The network balance was defined as the rate between the average excitatory and inhibitory synaptic currents, 〈〈Iexc〉N〈Iinh〉N〉t, in which 〈〉N stand for average among neurons and 〈〉t average on time. White squares indicate the two different parameter sets used in our simulations (τe = τi = 5 ms for AI Network, and τe = 1 ms, τi = 7.5 ms for PING Network). C: Same as B but calculated for the FS population. D: Population frequency autocorrelation of RS (green dots) and FS population (blue dots) neurons of the two used parameter sets. Solid lines indicate the damped cosine fitted function. (TIF) [file pcbi.1009416.s001.tif]

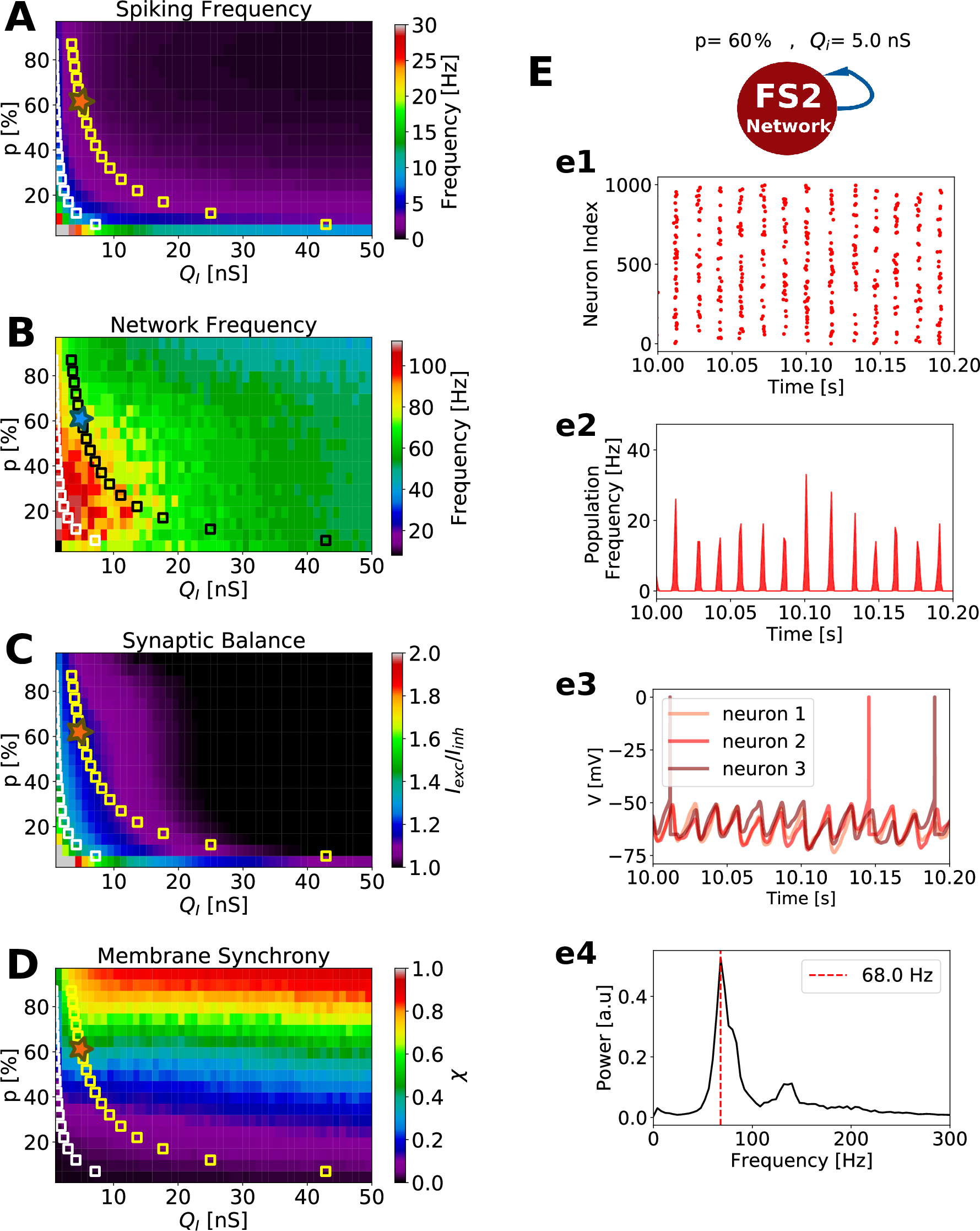

Supplement: S2 Fig — The network connectivity (p) vs. inhibitory synaptic strengths (Qi) parameter space of the Gamma Network are displayed as color-plots. A: Average spiking frequency. B: Network oscillation frequency. C: Network balance: rate between the average excitatory and inhibitory synaptic currents, 〈〈Iexc〉N〈IInh〉N〉t, in which 〈〉N stand for average among neurons and 〈〉t average on time. D: Membrane Potential Synchrony (χ), calculated by means of the equation: χ2=σV21NΣiNσVi2, in which V(t)=1NΣiNVi(t), σV2=〈[V(t)]2〉t-[〈V(t)〉t]2 and σVi2=〈[Vi(t)]2〉t-[〈Vi(t)〉t]2. The set of parameter which allowed Gamma Network to oscillate in the Gamma range are indicated by a star symbol. The white and yellow curves depict parameter choices in which the product between p and Qi are the same. The yellow curve indicates all parameters equivalent to a choice of p = 60% and Qi = 5 nS (Qi′=3/p′), while the white curve indicates all parameters equivalent to a choice of p = 10% and Qi = 5 nS (Qi′=0.5/p′), like it is usually used in other works [30]. Every point in each graph is given by the average output of 10 simulations of 5 seconds each. In this simulations each neuron of the Gamma Network received 400 independent and identically distributed excitatory Poissonian spike trains with a spiking frequency μExt = 5 Hz and a synaptic strength of QExt = 1 nS that decayed with synaptic time constant of τE = 5 ms. E: Network activity for the parameters indicated with a start in A, B, C and (p = 60% and Qi = 5 nS). The raster plot of the whole network (e1), the population frequency (e2), the membrane potential of 3 randomly chosen neurons (e3) and the power spectrum of the population frequency (e4) are indicated. The population frequency is calculated as the total number of spikes (spikes of the whole network) in a time bin of 1 ms, divided by the duration of this time bin. Because of the exclusive presence of inhibitory neurons and its high level of recurrent inhibition, this network is capable of generating [file pcbi.1009416.s002.tif]

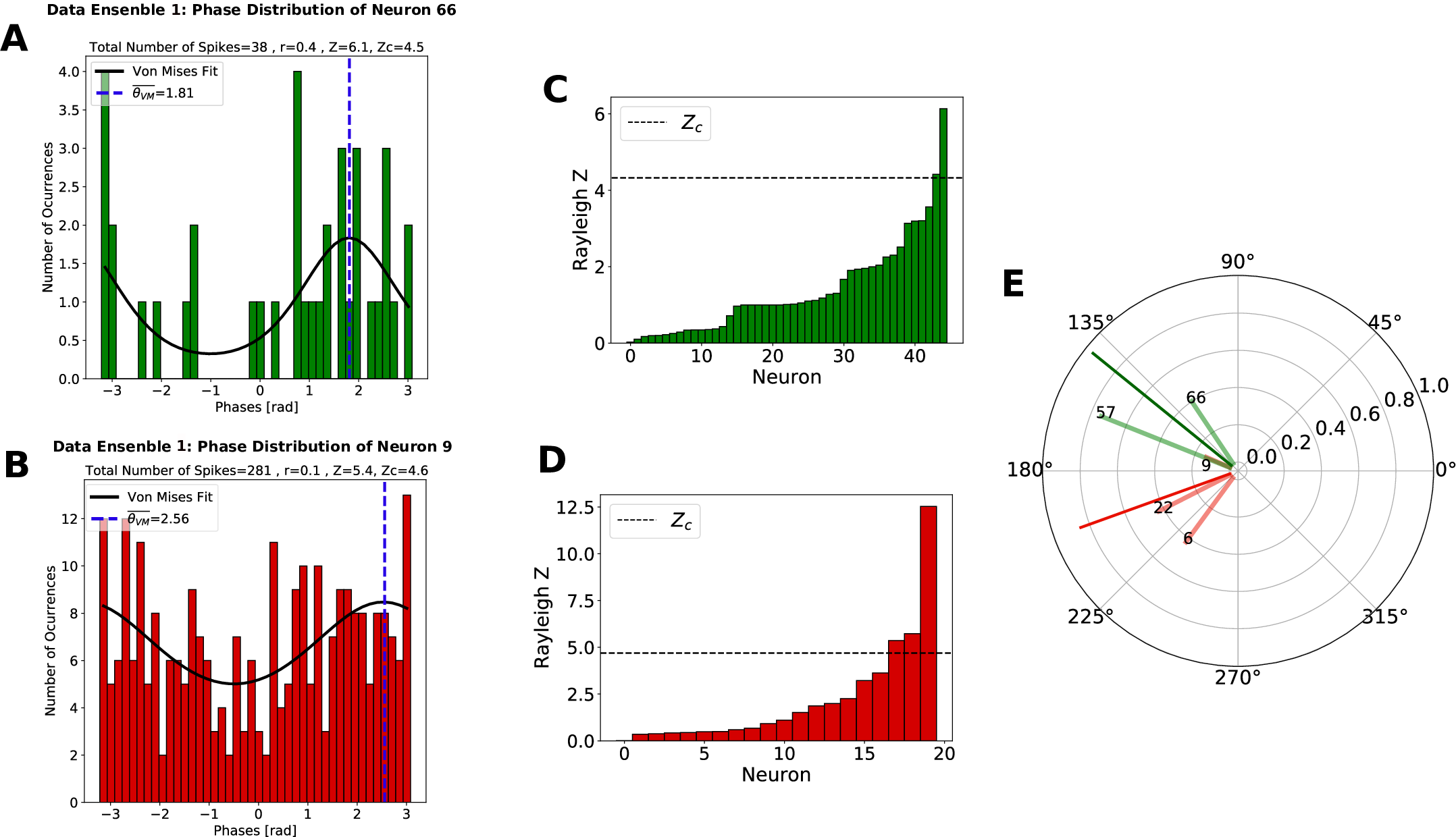

Supplement: S3 Fig — A and B: Phase distribution of two randomly picked cells from the human recordings (Data segment 1): one excitatory (A, green) and one inhibitory (B, red). The phase distribution of each cell was fitted to a Von Mises curve, which allowed the estimation of its preferred phase θVM¯. The phase distribution of each neuron was tested for circular uniformity using a Bonferroni-corrected Rayleigh test [36, 37]. C and D: Rayleigh Z calculated for all recorded neurons: excitatory (C, green) and inhibitory (D, red). A neuron was considered phase-locked if the circular uniformity at P < 0.01, (Z > Zc) could be rejected. In these plots, neurons were ordered according to their Z value and not according to their original indexes. E: Preferred phases, θVM¯, of each phase-locked cell, displayed in polar graph representation. Dark colored vectors indicate the average phase among each neuron type and Δθ the phase difference among RS and FS. Data segment 1 presented 22 minutes of recordings, containing 9 seconds of Gamma activity. (TIF) [file pcbi.1009416.s003.tif]

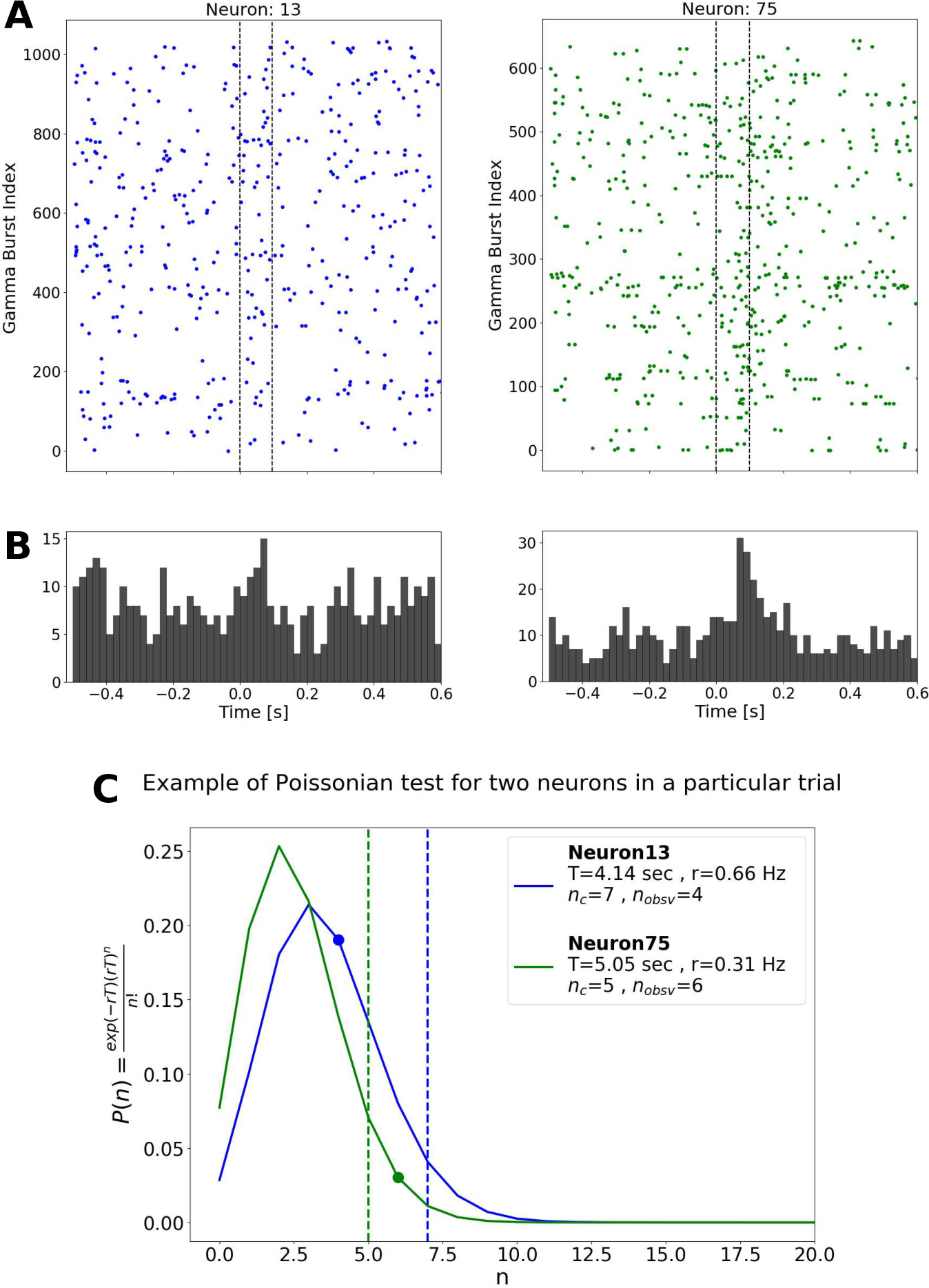

Supplement: S4 Fig — A: Activity of two randomly picked cells during several Gamma bursts: neuron 13 (inhibitory, left) and neuron 75 (excitatory, right). The graphs display the firing patter around Gamma bursts (indicated by the black doted lines). Each point corresponds to one spike in the correspondent tuple of time and burst ID (y-axis). B: Histogram computing the distributions of all spikes inside all Gamma bursts of neuron 13 (left) and neuron 75 (right). C: Exemplification of firing rate change statistical test. The Poissonian distribution of these two neurons is constructed based on their average firing rate calculated outside of Gamma bursts. The critical number of spikes nc, indicated by the dotted lines, is calculated based on the Percent Point Function of the respective Poissonian Distribution for a period T, with an 95% Interval of Confidence. The observed number of spikes nobsv is depict as a dot over the curve. According to this procedure, only neuron 75 is considered to increase its firing, since nobsv > nc. (TIF) [file pcbi.1009416.s004.tif]

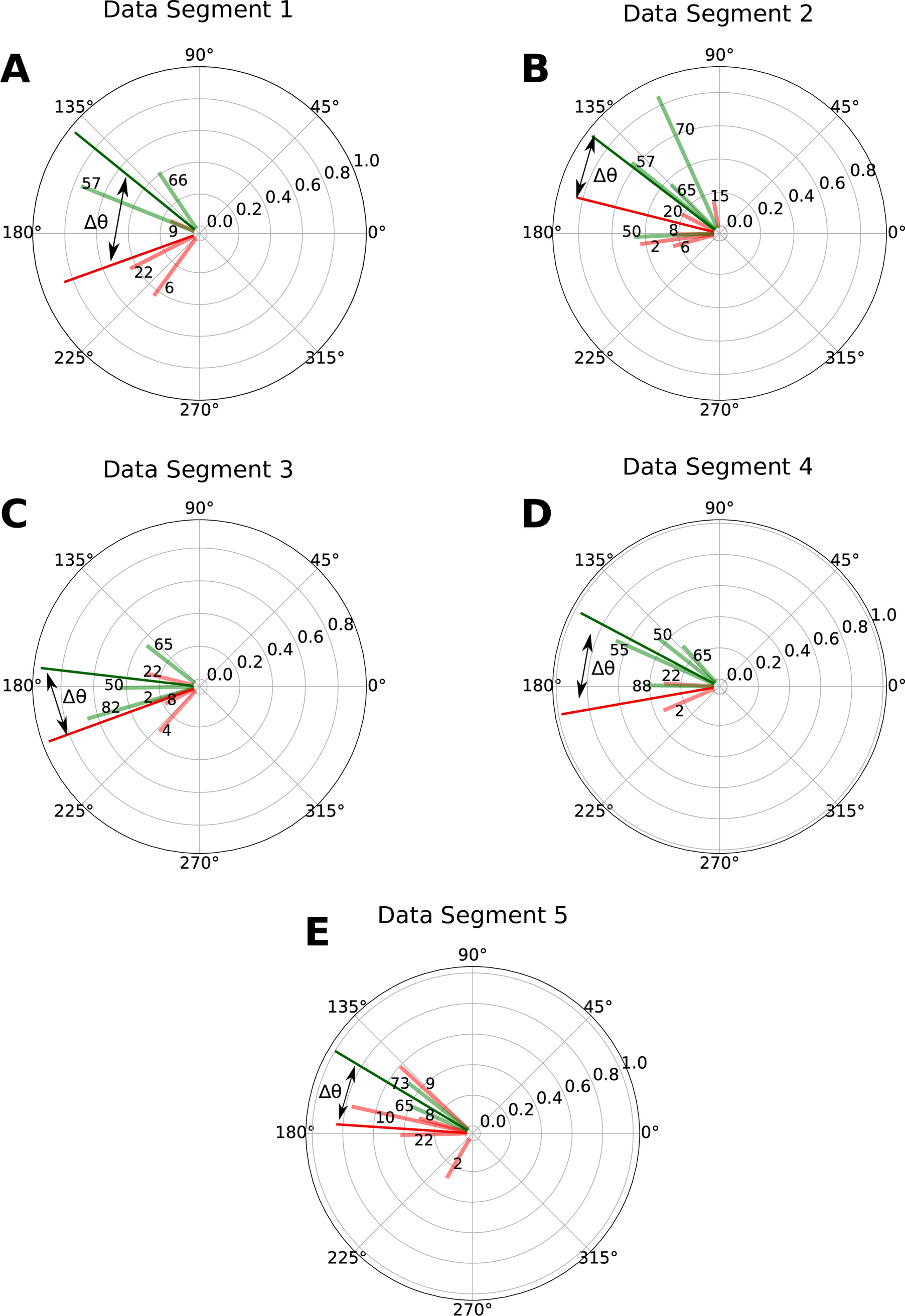

Supplement: S5 Fig — A: Data segment 1—containing 22 minutes of recordings and 9 seconds of total Gamma activity. B: Data segment 2—containing 43 minutes of recordings and 14 seconds of total Gamma activity. C: Data segment 3—containing 28 minutes of recordings and 16 seconds of total Gamma activity. D: Data segment 4—containing 26 minutes of recordings and 13 seconds of total Gamma activity. E: Data segment 5—containing 16 minutes of recordings and 11 seconds of total Gamma activity. The preferred phases of each phase-locked cell are displayed in polar graph representation. Phases were calculated from −π to π. The vector size gives a measure of the phase distribution of each cell. Big amplitude vectors indicate very concentrated distributions while small amplitude vectors indicate less concentrated ones. The color of each vector encodes the type of the cell of whom it represents the phase: red (FS), and green (RS). Cell number IDs are indicated. Dark colored vectors indicate the average phase among each neuron type and Δθ the phase difference among them. (TIF) [file pcbi.1009416.s005.tif]

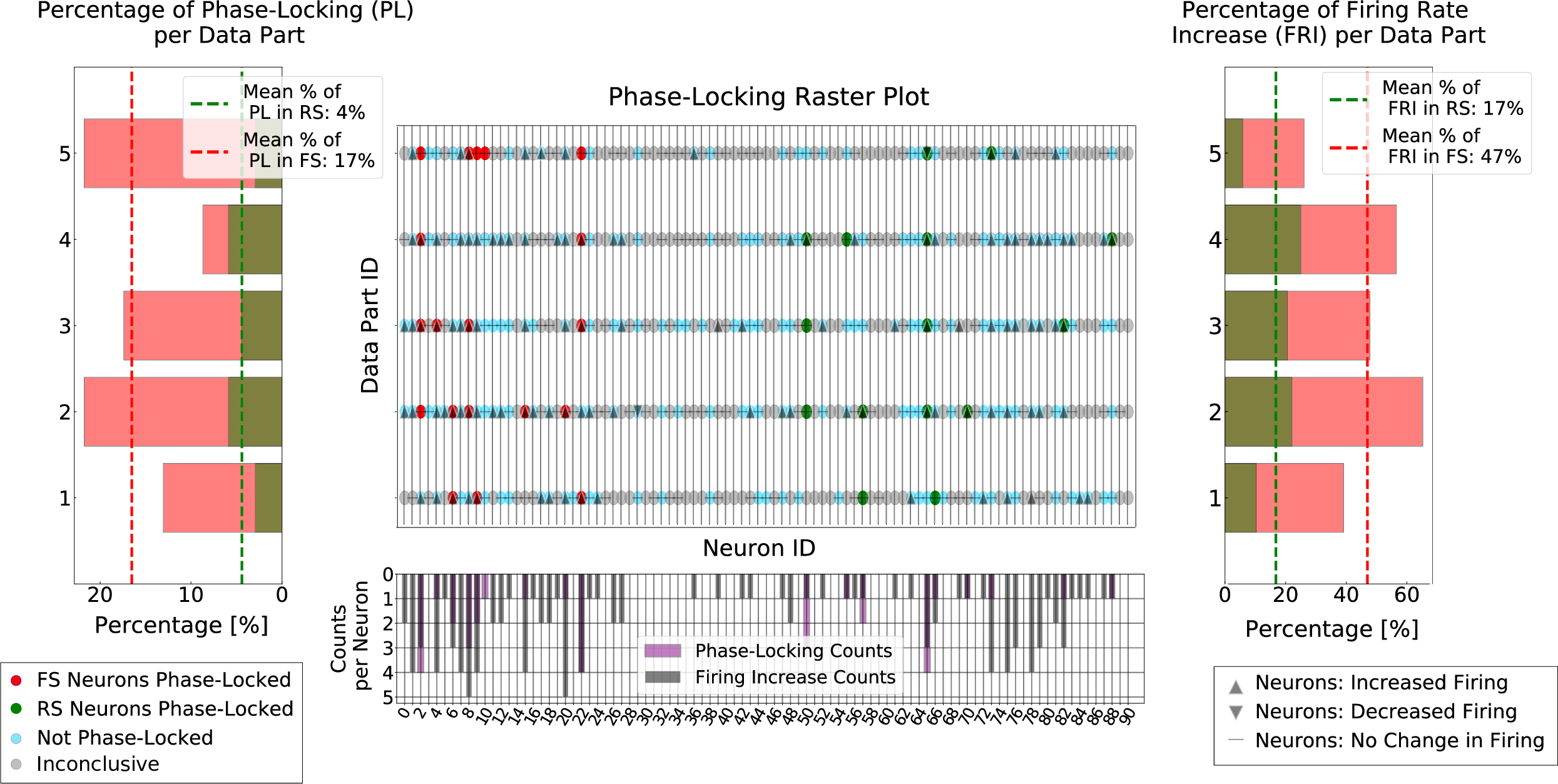

Supplement: S6 Fig — The middle panel represents each cell by a circle in each of the 5 data segments. FS and RS phase-locked cells are depicted respectively as red and green circles, while not phase-locked or inconclusive (with respect to phase locked) cells of both types are depicted as blue and gray circles respectively. Superposed to each cell circle, pointing up and down triangles were added to indicate if the cell increased (△) or decreased (▽) its firing. If the cell didn’t change its firing significantly a minus sign (-) was added. Side box plots indicate, on the left, the percentage of phase-locked FS (red) and RS (green) cells in each of the 5 data segments, and, on the right, the percentage of firing rate increase. Dotted lines indicate the average value (phase-locking level: left and firing rate increase: right) between the 5 data segments. The bottom box plot depicts the superposed counts of phase-locking or firing rate increase behavior of each individual cell, computed in the 5 data segments. (TIF) [file pcbi.1009416.s006.tif]

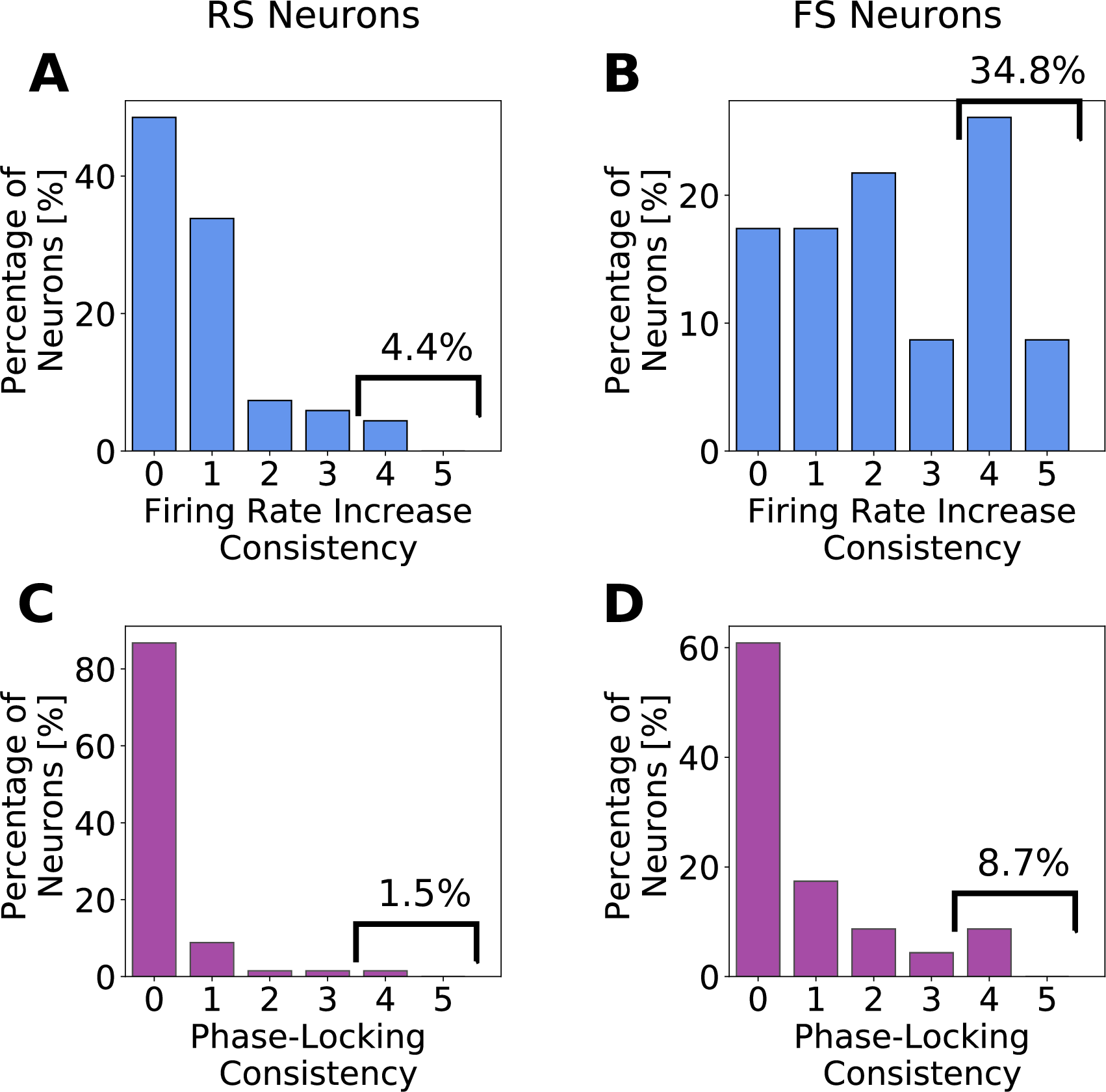

Supplement: S7 Fig — Distributions of consistency indexes among the recorded neurons with respect to to firing rate increase are displayed respectively in A and B for RS cells and FS cells, while C and D display the consistency indexes distribution of phase-locking for RS and FS. (TIF) [file pcbi.1009416.s007.tif]

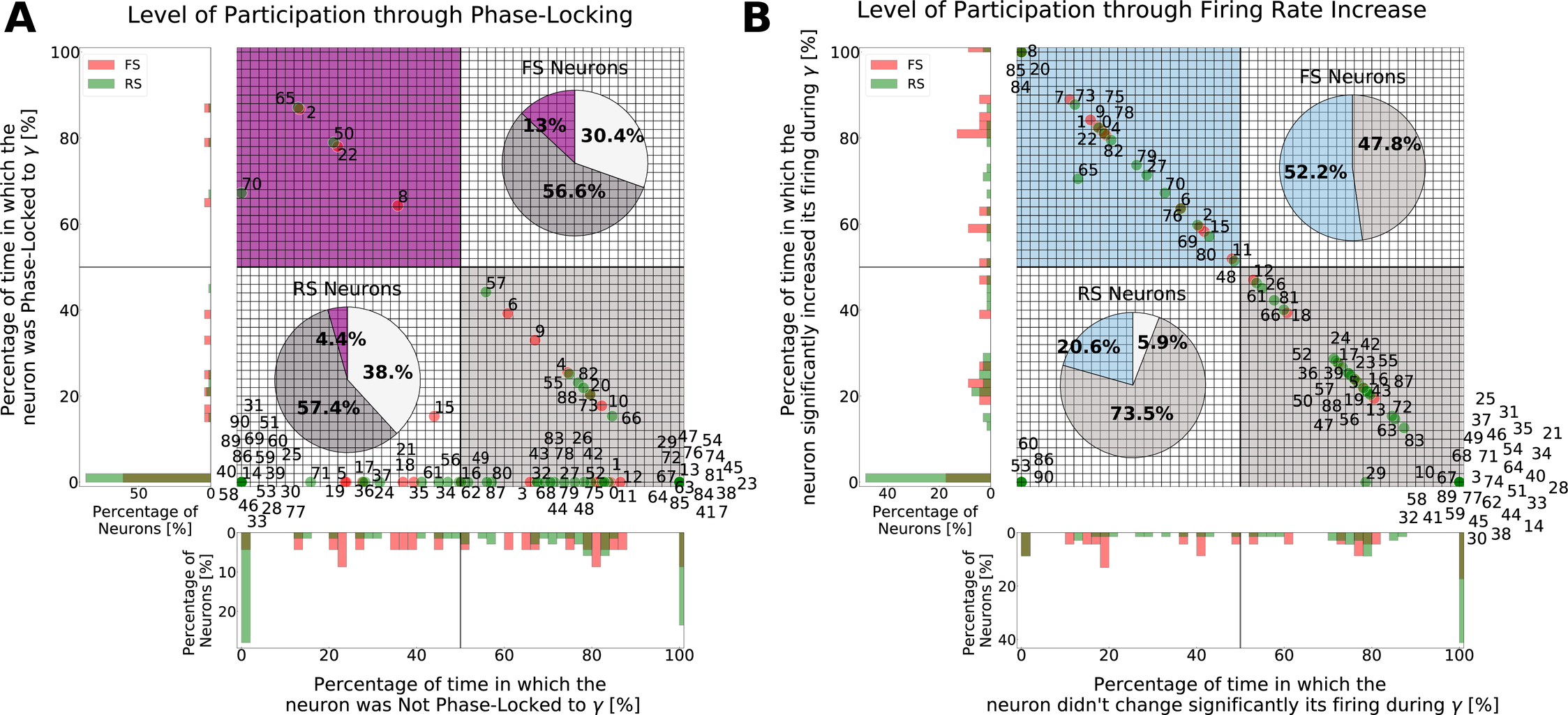

Supplement: S8 Fig — The activity of each neuron inside and outside Gamma bursts in all 5 data segments were quantified. Taking into account that each data segment had a different duration, containing a different total Gamma duration, and that some neurons were silent in some data segments, each neuron was analyzed individually, taking into account the percentage of the total amount of time in which the neuron was active. A: Phase-locking time distribution. The grid plot in the middle displays the amount of time (with respect to the total recording time) in which each neuron was considered phase-locked (A, y axis), and the the amount of time in which each neuron was considered not phase-locked (A, x axis). RS neurons are depicted in green and FS neurons in red, together with their ID number. Neurons lying outside of the diagonal are neurons of whom statistical analysis was inconclusive at some data segments, due to the reduced number of spikes. At the top left corner, lie neurons that were always considered phase-locked, while neurons that were never considered phase-locked are placed at the bottom right corner. Pie plots indicate the percentage of neurons that passed at least 50% of the total time being either phase-locked or not phase-locked (neurons that fall inside of the colored quadrants) and the neurons lying on the left white quadrant. B: Same analysis as A but displaying the firing rate change time distribution. This analysis indicates that only a small percentage of neurons passed at least 50% of the total time being either phase-locked (RS: 4.4%, FS: 13%) or increasing its firing (RS: 20.6%, FS: 52.2%). Moreover, even though no cell was 100% of the time phase-locked to Gamma, some cells were 100% of the time not phase-locked to Gamma (RS: 22.1%, FS: 13%) and others never increased their firing (RS: 41.2%, FS: 17.4%). (TIF) [file pcbi.1009416.s008.tif]

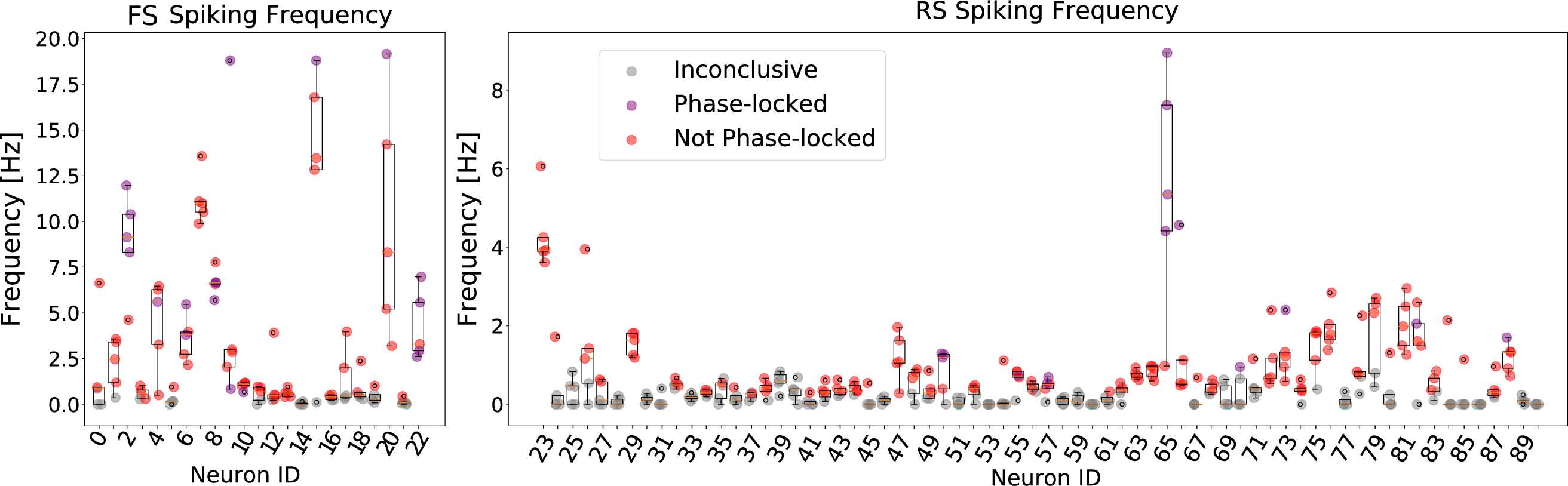

Supplement: S9 Fig — The average firing rate of each neuron in each of the 5 data segments (inside Gamma bursts) is depicted as a point in this graph (each neuron presents 5 points). The color of each point corresponds to the neuron classification with respect to phase-locking in the correspondent data segment (purple: phase-locked, red: not phase-locked and gray: inconclusive). The average firing rate inside Gamma bursts was calculated based on the total Gamma duration (recorded by the electrode, that also recorded the particular neuron, in the respective data segment) and the total number of spikes emitted by this particular neuron exclusively inside the Gamma bursts of the respective data segment. Cells classified as inconclusive are cells that spiked less then 5 times inside Gamma bursts, or cells whose electrode measured less then 1 second of Gamma bursts in the respective data segment. FS neurons are depicted on the left and RS neurons on the right. Box plots referent to each neuron distribution are added to help in the visualization (regardless of the reduced number of points). The box extends from the lower to upper quartile values of the data, with a line at the median. The whiskers extend from the box to show the range of the data. Flier points are those past the end of the whiskers and are depicted with black circle together with the color point. This graph illustrates the fact that phase-locking and not phase-locking behaviors are observed both in cells with high and low firing rates. (TIF) [file pcbi.1009416.s009.tif]

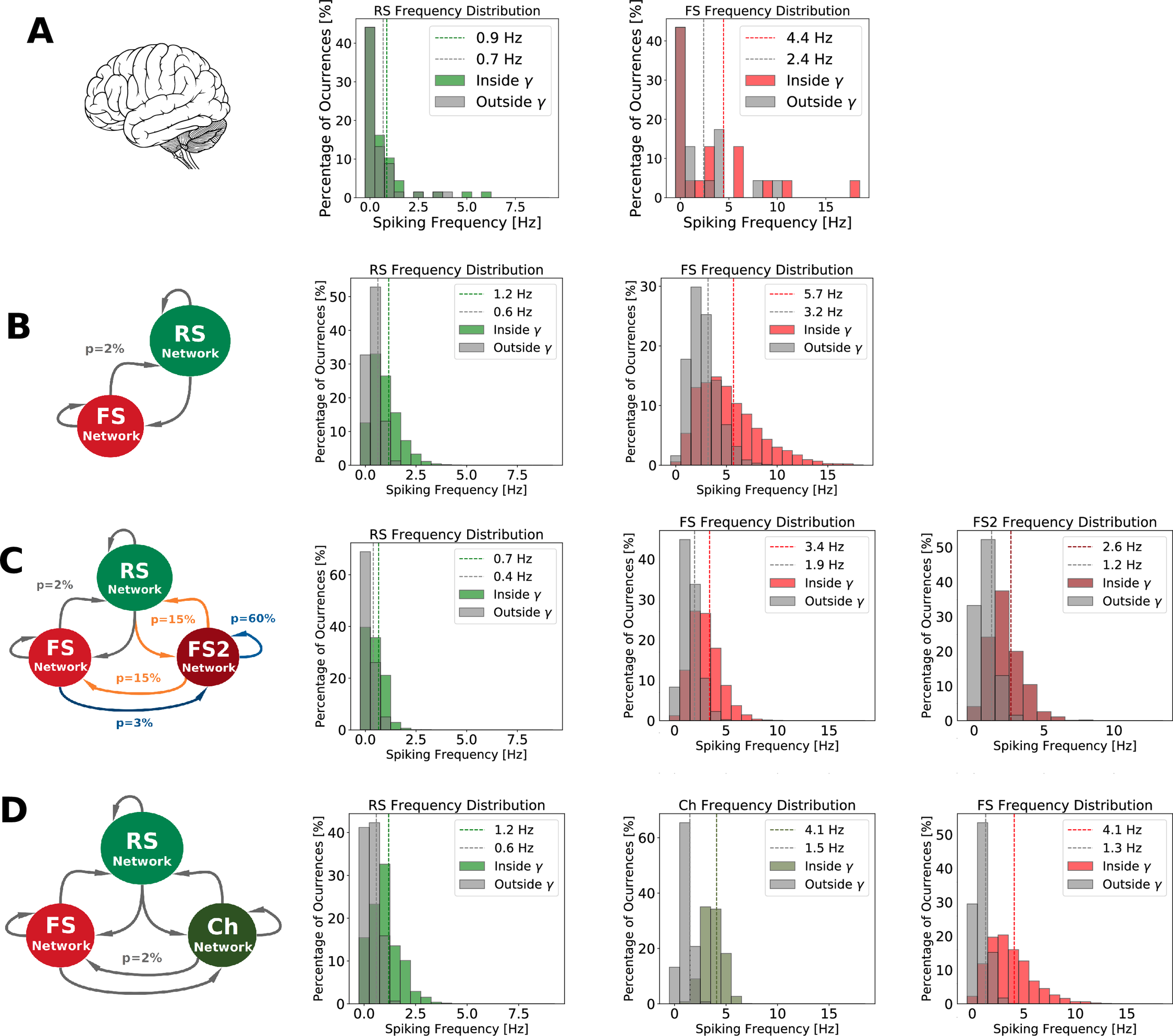

Supplement: S10 Fig — Firing rate distributions of different neuron types (inside and outside Gamma bursts) are depicted in A, B, C and D for each studied system. A: Human recordings. B: PING Network. C: ING Network and D: CHING Network. Average firing rates of each cell type is indicated by the dotted line. (TIF) [file pcbi.1009416.s010.tif]

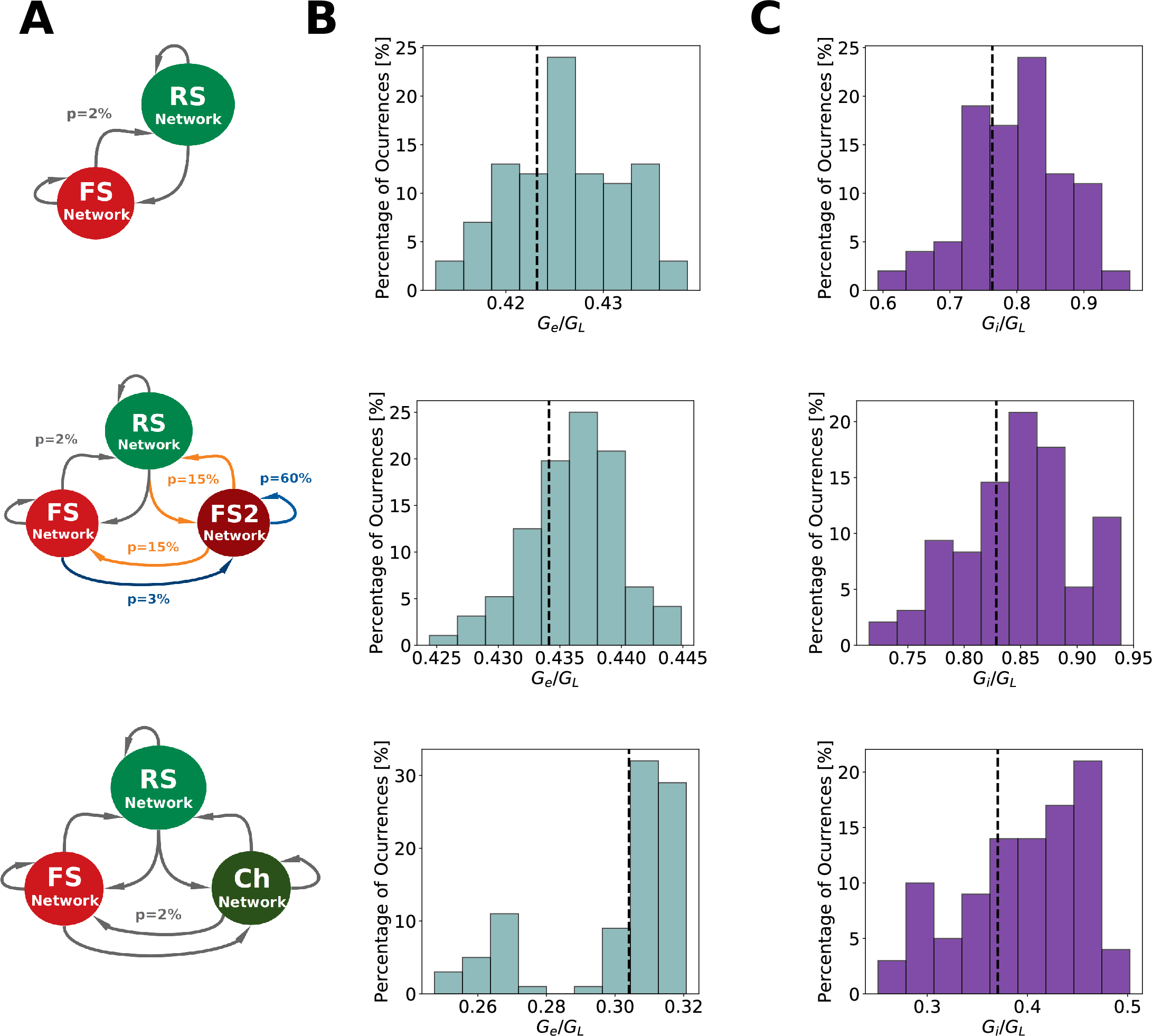

Supplement: S11 Fig — A: Illustration of the analyzed system: PING Network, ING Network and CHING Network. B: Ratio between excitatory conductance (Ge) and leakage conductance (GL). C: Ratio between inhibitory conductance (Gi) and leakage conductance (GL). Averages are indicated by the dotted line. The distributions fall inside of the physiological range observed experimentally [87]. (TIF) [file pcbi.1009416.s011.tif]

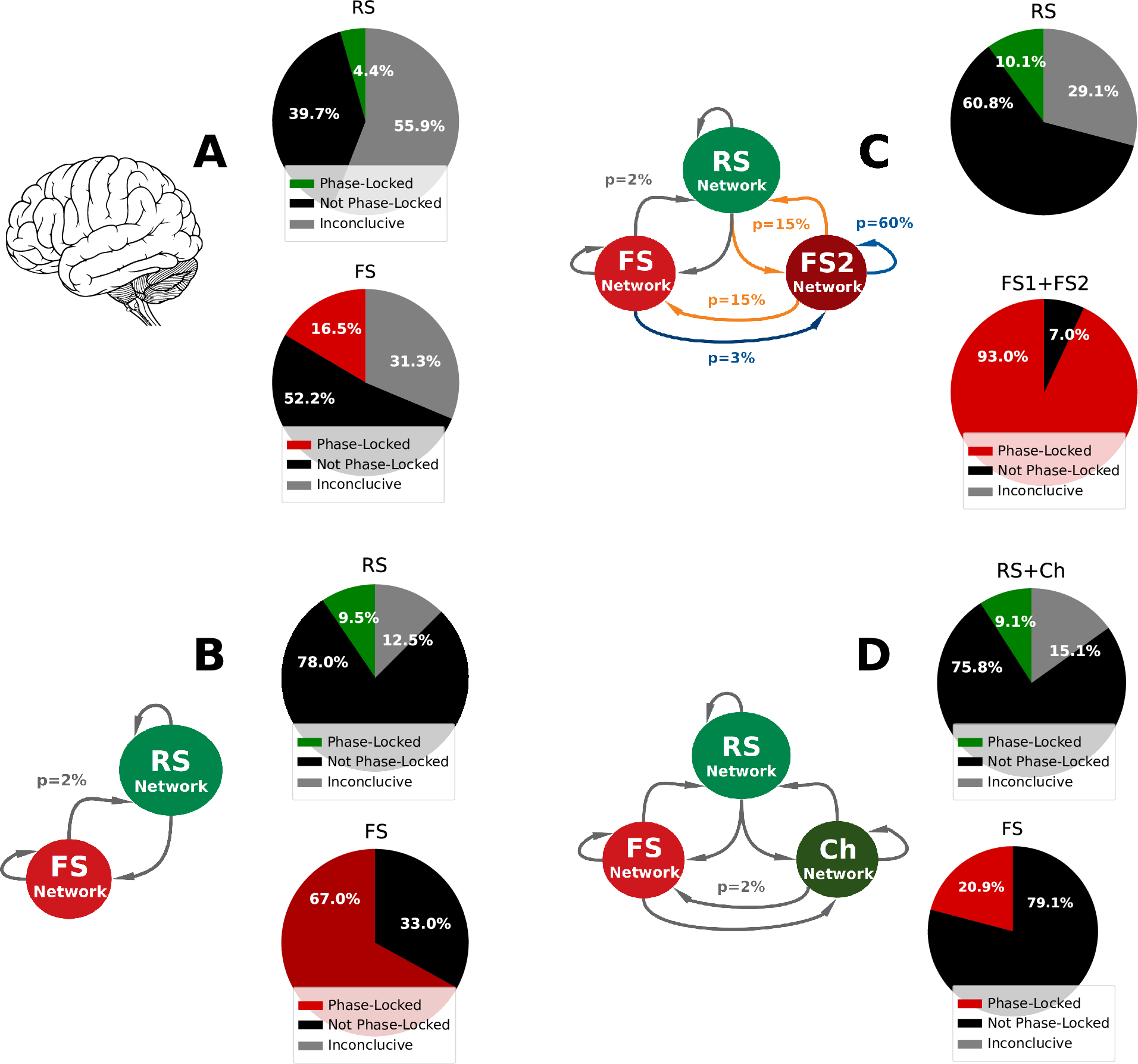

Supplement: S12 Fig — The average level of phase-locking is defined as the averaged percentage of cells in the network considered to be phase-locked, across the 5 segments of data recorded. The analysis was done separately for excitation and inhibition. A: Human Data recordings, B:PING Network, C: ING Network and D: CHING Network. The percentage of cells signaled as inconclusive relates to cells in which the number of spikes inside Gamma burst were too small to allow statistical significant phase-locking. (TIF) [file pcbi.1009416.s012.tif]

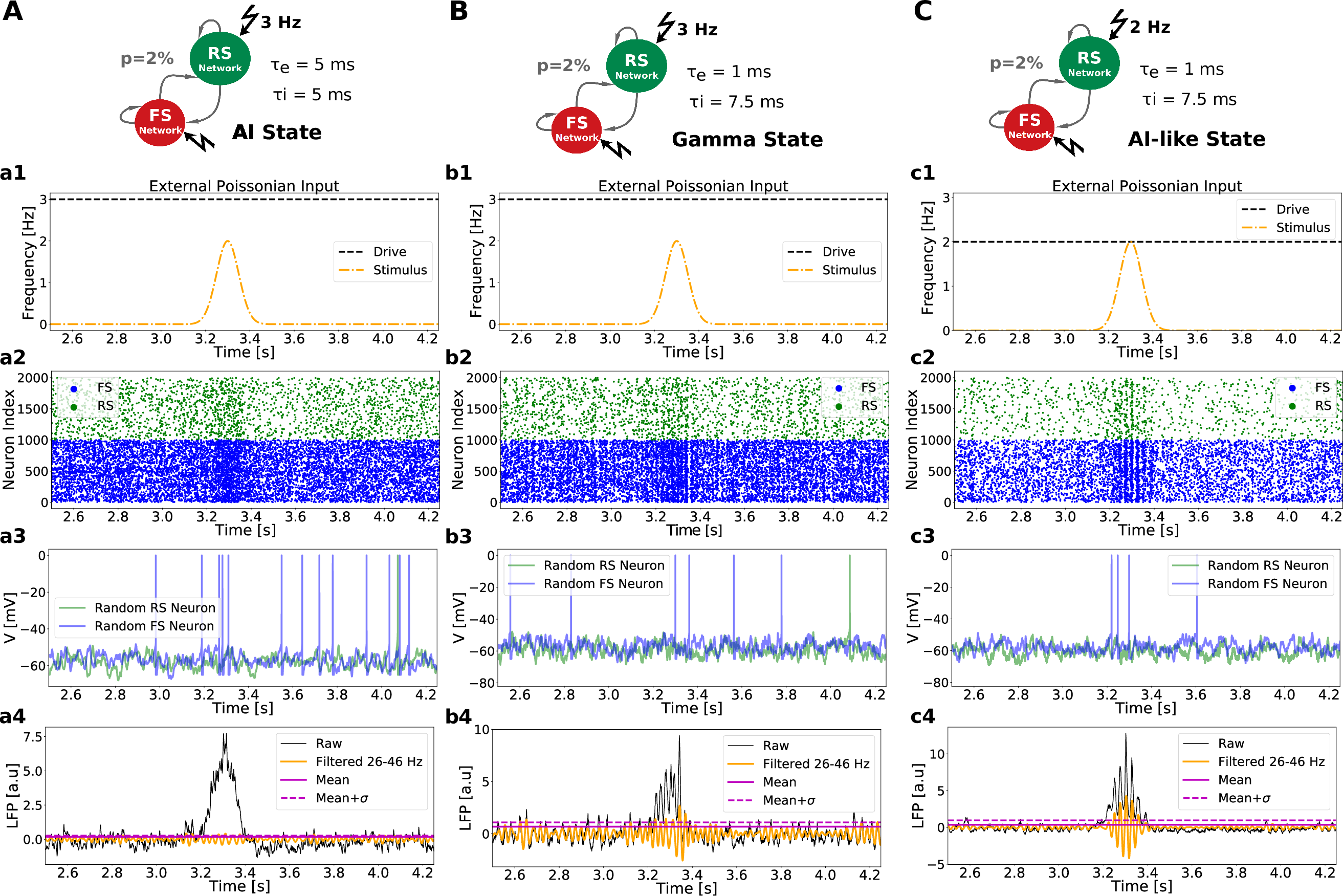

Supplement: S13 Fig — A: AI network receiving a Poissonian drive of 3Hz. B: PING network receiving a Poissonian drive of 3Hz (inducing Gamma). C: PING network receiving a Poissonian drive of 2Hz (not inducing Gamma). In addition to the drive each network received a Gaussian stimulus of 2Hz pick and a standard deviation of 50 ms. The drive and stimulus are depicted in each case in a1, b1 and c1. The raster plot of each network during the stimulation is depicted in each case in a2, b2 and c2. The membrane potential of 3 randomly picked neurons are depicted in each case in a3, b3 and c3. The raw and the filtered simulated LFP are depicted in each case in a4, b4 and c4. (TIF) [file pcbi.1009416.s013.tif]

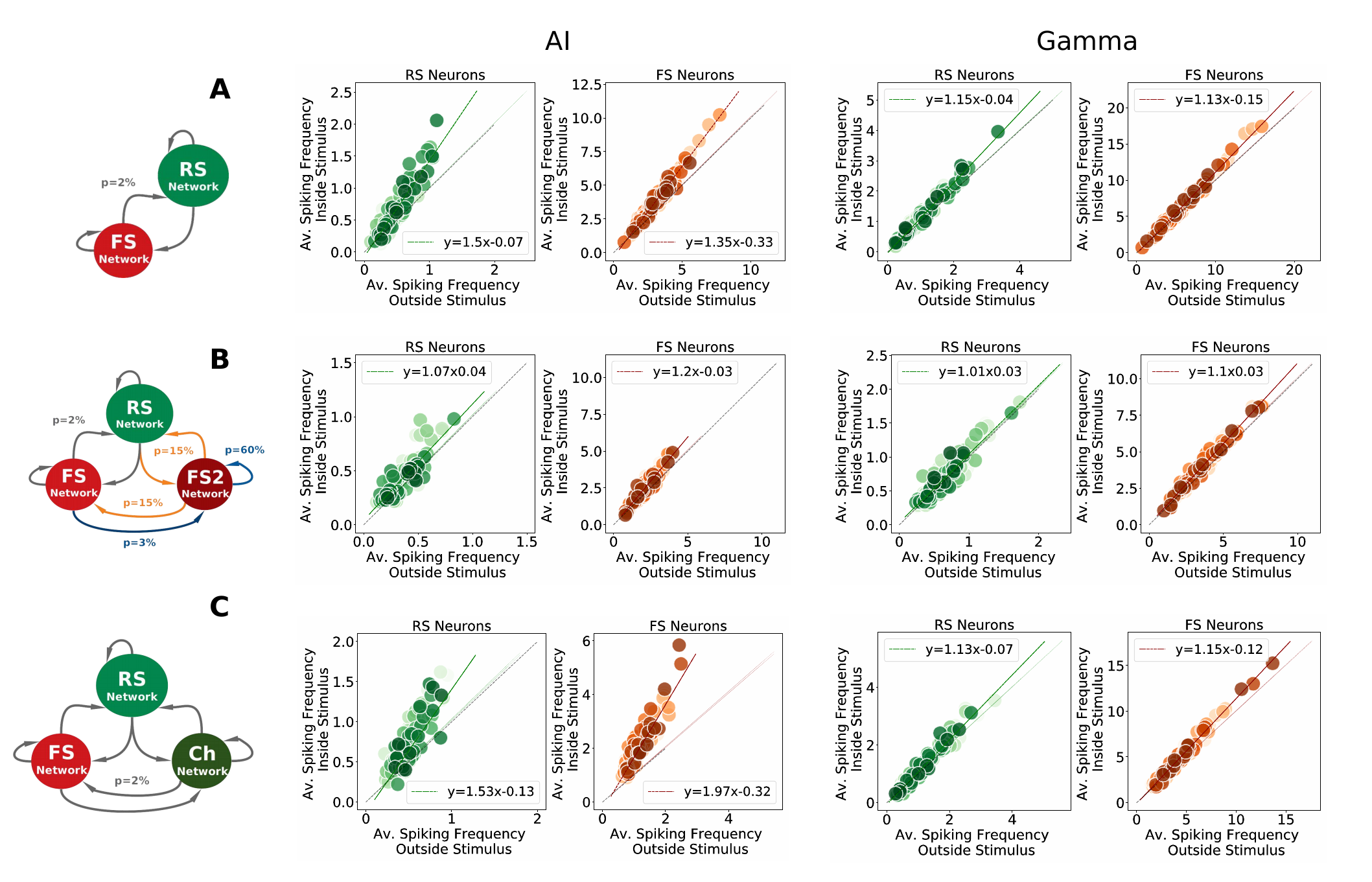

Supplement: S14 Fig — A: PING Network. B: ING Network. C: CHING Network. To estimate the individual cell responsiveness, we calculated the average spiking frequency of each cell inside (y-axis) and outside stimulus (x-axis) during AI-like states (left) and Gamma states (right). RS cells are displayed in green and FS cells in red. In each plot the linear regression from the points is depicted with the identity. We observe that all cells follow the same rule of responsiveness (proportional to their firing outside the stimulus). No difference can be seen between the responsiveness of neurons classified as Gamma participating and the Gamma non-participating cells. (TIF) [file pcbi.1009416.s014.tif]

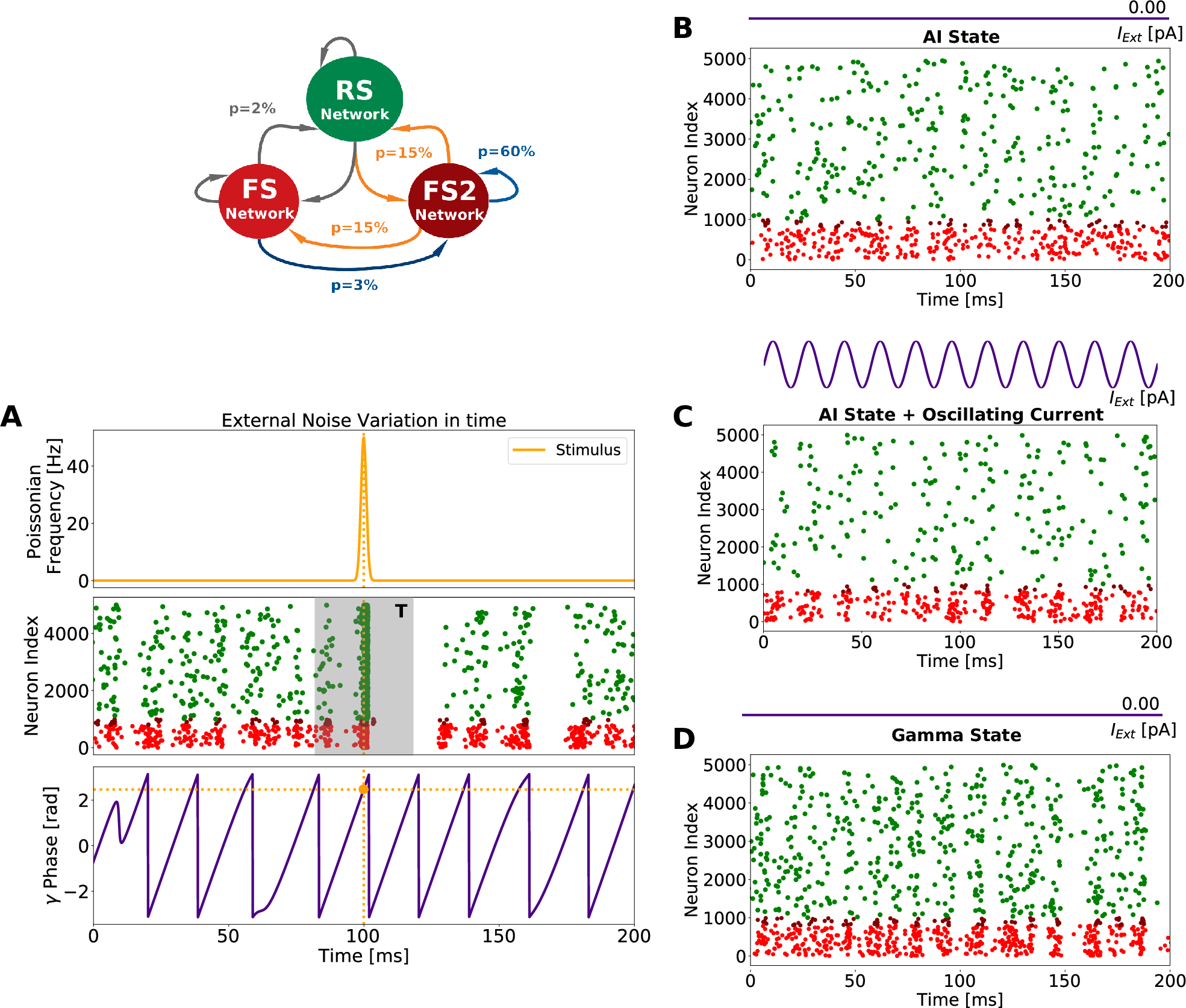

Supplement: S15 Fig — A: Protocol scheme in ING Network when it displays Gamma oscillations (45-65 Hz). Top: stimulus used to measure network phase-dependent response. The stimulus consisted of fast Gaussian fluctuation (standard deviation of 1 ms) which modulated the firing rate of the external Poissonian spike trains injected into network from 0 to 50 Hz. Middle: Raster plot indicating the network response to the Gaussian stimulus. The network responsiveness was calculated according to Eq 6, in a time window T = 18ms (shaded gray area). Bottom: Gamma oscillation phase around the the stimulus pick. The phase at the time the stimulus was applied is indicated. The Phase-dependent network responsiveness was measured in three different network states: B: AI state (Poissonian noise = 2Hz, no external current). C: AI-modulated states (Poissonian noise = 1Hz, with sinusoidal external current). D: Gamma state (Poissonian noise = 3Hz, no external current). Items A, B and C display the Raster activity of ING Network without the Gaussian stimulation. Only 20% of network is shown. (TIF) [file pcbi.1009416.s015.tif]

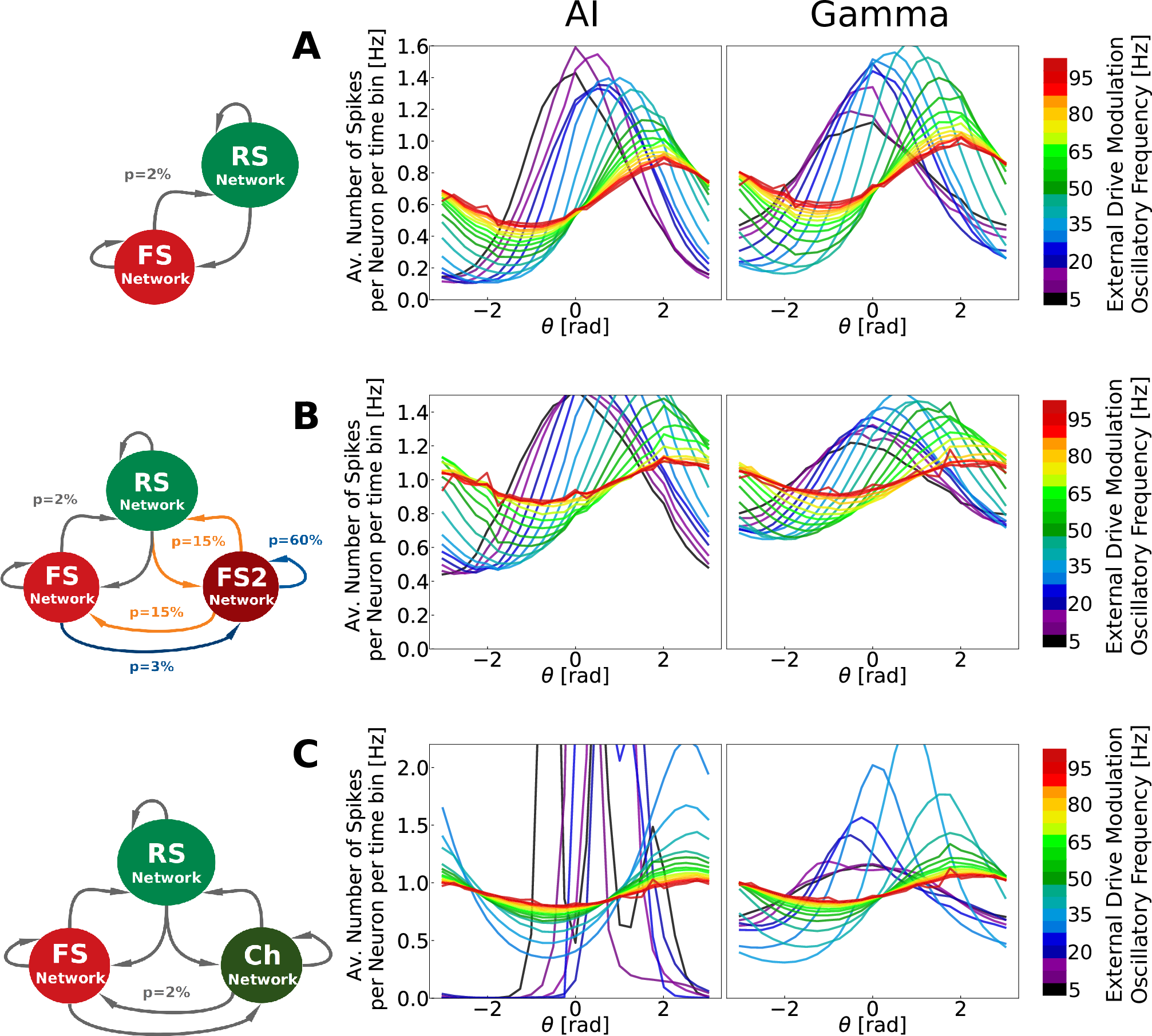

Supplement: S16 Fig — A: Resonant properties of PING Network. B: Resonant properties of ING Network. C: Resonant properties of CHING Network. The curves displayed in B, C and D depict, for each oscillatory frequency (color scheme) the amplitude (average number of spikes per neuron per time bin) as a function of the oscillation phase, during Gamma and AI-like states. All values were normalized by the average firing inside of each state to exclude the state dependent firing rate level (which is higher on Gamma). Δnoise = 0.5 Hz in all network models but μnoise varied in each case. For AI, in PING and ING Networks μnoise = 2 Hz and in CHING Network μnoise = 1 Hz, while for Gamma, μnoise = 3 Hz in in PING and ING Networks and μnoise = 2 Hz in CHING Network. (TIF) [file pcbi.1009416.s016.tif]

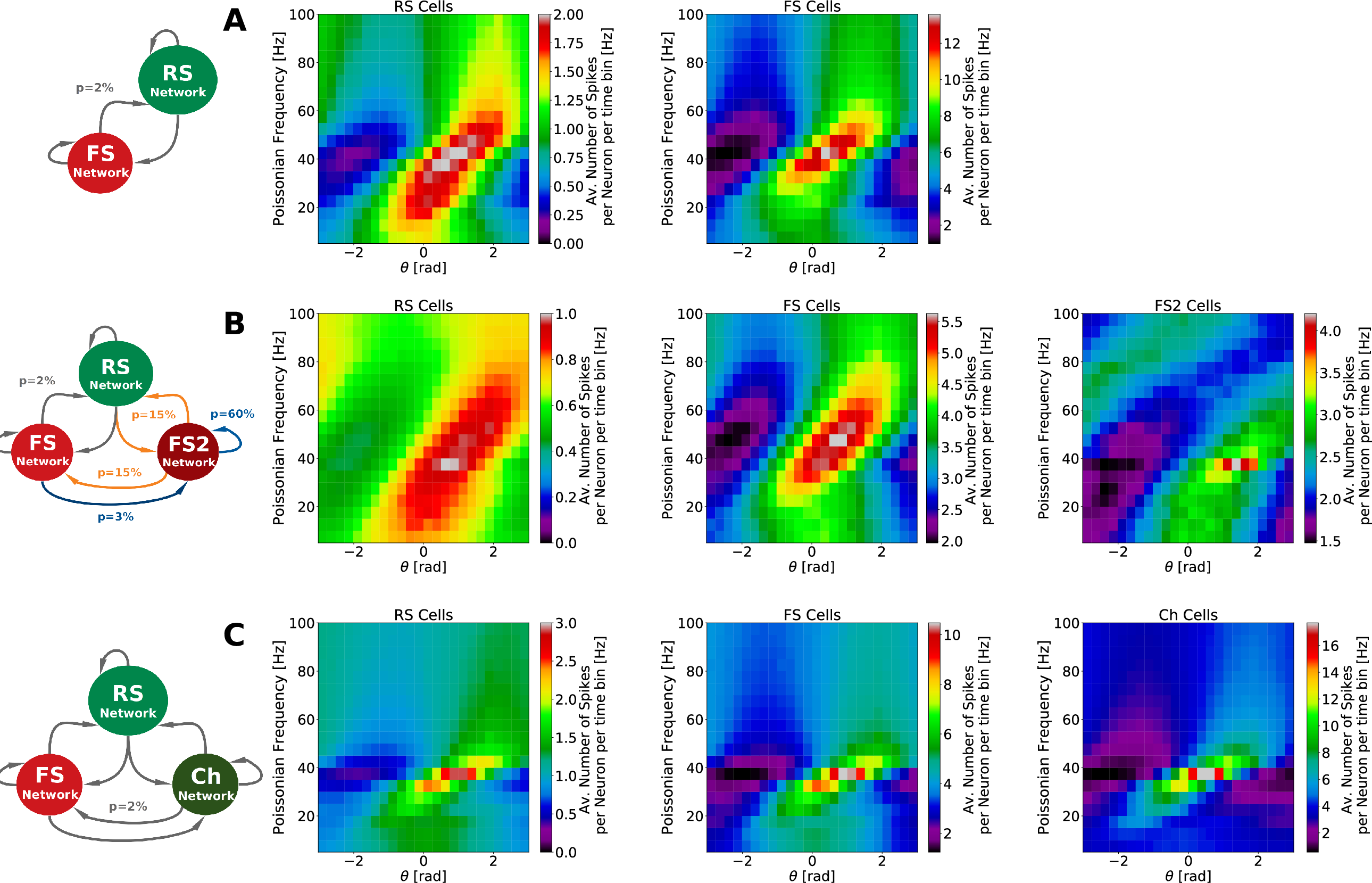

Supplement: S17 Fig — A: Resonant properties of PING Network. B: Resonant properties of ING Network. C: Resonant properties of CHING Network. The color maps displayed in A, B and C depict, for each oscillatory frequency and oscillation phase, the average number of spikes per cell type (RS, FS, FS2 or Ch) and time bin, during Gamma state. Differently than Fig 9 no normalization was applied. Δnoise = 0.5 Hz in all network models but μnoise varied in each case. In PING and ING Networks μnoise = 3 Hz and in CHING Network μnoise = 2 Hz. (TIF) [file pcbi.1009416.s017.tif]
