# Supplementary material for: Integration, coincidence detection and resonance in networks of spiking neurons expressing Gamma oscillations and asynchronous states
Source: PLoS Comput Biol. 2021 Sep 16;17(9):e1009416. doi: 10.1371/journal.pcbi.1009416 (PMC8478196; doi:10.1371/journal.pcbi.1009416)
Supplement: Attachment — Submitted filename: Response_to_reviewers.pdf [file pcbi.1009416.s018.pdf]

Reply to the Reviewers

Reviewer #1:

The manuscript describes computer simulations of 3 neural network models. The authors compare the dynamics of their models during gamma oscillation bursts to some previously published human recordings data and then test the model activity responses under different external stimuli. The simulations appear to have been competently performed. However, I do not find particularly striking the main conclusion that the networks are less responsive during gamma oscillation bursts. It should in any case be made more convincing (point 4 below).

At the technical level, several points should be addressed :

***1)** In the comparison between the different networks and the data, I did not find the value of spiking rates of the FS2 neurons (the only information that I found is Fig S1A where it appears that FS2 cells fire at a very low frequency -in this figure Panel A and B seems to be inverted as compared to the caption). I would be helpful to give it, for instance to give separately FS and FS2 histograms in Fig S7).*

Answer: You are right, panels A and B of Fig S1A were inverted in comparison to the caption. This was corrected and spiking frequency distributions were plotted separately for FS and FS2 in Figure S7. We used the opportunity to also include the distributions of RS and Ch separately.

***2)** A related point is that an oscillation of 50Hz appears quite low for an ING network. For instance, given the results of ref.36, one would expect a pure ING to be more suitable for the description of 100-200Hz oscillation. Is this related to the low firing rate of the FS2 neurons? How sensitive is this oscillation frequency? Comments from the authors on this point would be appreciated.*

Answer: The *ING network* is able to generate Gamma rhythms at 50 Hz thanks to the inclusion of the *Gamma network* composed of FS2 neurons, which are highly connected. Figure S1 shows that, like in reference 36, if small probabilities of connection are used, the network will oscillate at high frequencies (100-200Hz). If on the other hand high connectivity patterns are used (60 % in our case) slower rhythms are possible (~70 Hz). To allow a better understanding, we added to this figure the raster, the membrane potential of 3 randomly selected FS2 cells and the population frequency and its power spectrum, for the selected parameters indicated with the star. When the *Gamma network* is merged with the *AI Network* to form the *ING Network* the frequency of the oscillation slows down further, reaching ~55 Hz as shown in Figure 4.

3) *I am somewhat confused by the authors' convention and discussion of the relative firing phases and order of the different populations I the oscillations. I would expect the phase to be proportional to time, so that a population with the larger phase fires after one with a smaller phase i.e. that time would go in counterclockwise direction in the circular representation of Fig 6. For instance, in Fig.6 from the diagram I would expect the FS population (red) to fire before the RS one (green). However, reading from the text, the converse is described by the authors. Please clarify.*

Answer: You are right, this aspect was not very well explained. As you can see in Supplementary Figure S3, phases were calculated from -pi to pi. In this way, neurons with negative phases should be interpreted as spiking preferentially before than neurons with positive phases.

The plot in Figure 6 is a polar plot. Normally as you said, if the phases are measured from 0 to 2pi we should interpreted the graph counterclockwise with time. But since the phases were measured from -pi to pi, this plot should be interpreted clockwise with time.

To clarify, we added a sentence in the methods section "Spike-LFP phase-locking" and one in the legend of Figure 6.

4) *That the network are less sensitive to external inputs during gamma bursts appears to be the central result of the paper. However, the external input is provided on top of the input creating the gamma burst. Thus, one wonders whether this diminishes sensitivity is due to the gamma oscillation or simply to the nonlinear addition of two inputs. It would be good to compare with a parameter case in the AI regime where the first input does not create oscillation to try and discriminate the two effects.*

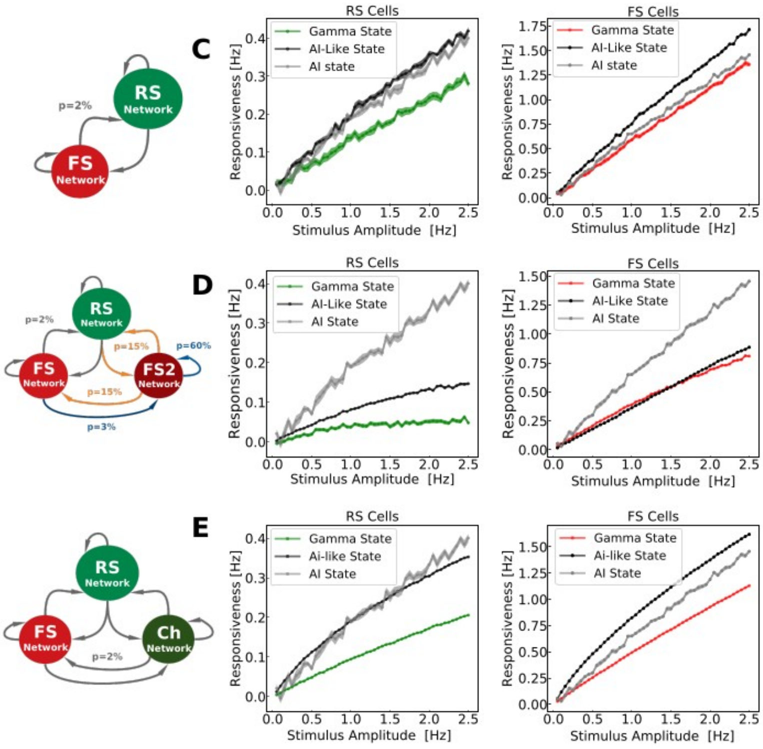

Answer:

This is an excellent question, but we think it was already addressed in the paper, but was insufficiently explained. In fact, the protocol described was referring to the case in which the responsiveness was measured during Gamma, the only case in which there is a drive fluctuation. During AI-like states the drive is constant and as such does not generate Gamma. We corrected the legend of the figure to make it more clear.

On the other hand, this question made us think about an important point that was also not well addressed. During AI-like states, the Gaussian stimulus was generating Gamma as can be seen in a new added supplementary figure S13. For this

reason, to be able to measure the real impact of Gamma oscillations on network responsiveness, we added to Figure 7 the responsiveness curves from the *AI-Network, in which no oscillation is generated, independently of the level of external drive. See legend on the edited article*.

The responsiveness of a network in a real AI-state (gray curve generated by the AI Network) is equal or higher to the AI-like responsiveness in each of the networks and is always higher then the Gamma state responsiveness in all cases, indicating that our result is consistent.

The *AI-Network*, regardless of the drive strength, can not generate oscillations because of the particular choice of time scales ($\tau e= \tau i= 5ms$), like it is shown in the supplementary figures S1 and S13 (added to the edited article). Like this, the responsive curve generated by this network is a good control to verify if the difference of responsiveness seen between AI-like and Gamma are due to the oscillation or merely due to the nonlinear addition of two inputs.

In the edited Figure 7, *AI-like* stands for the responsiveness curve generated by the respective network (PING, ING or CHING) when it is driven by a small frequency drive (2Hz for PING and ING, and 1Hz for CHING), *Gamma* stands for the responsiveness curve generated by the respective network (PING, ING or CHING) when it is driven by a higher frequency drive (3Hz for PING and ING, and 2Hz for CHING) and AI stands for the responsiveness curve generated by the *AI-network* when it is driven by an external drive of 3Hz. The AI-network present no Gamma oscillation even when stimulated by the extra Gaussian stimulus, while the PING, ING and CHING when working on the AI-like state start presenting Gamma when the Gaussian is applied. To show this fact clearly we produced an extra supplementary figure (S13) for the PING case copied bellow.

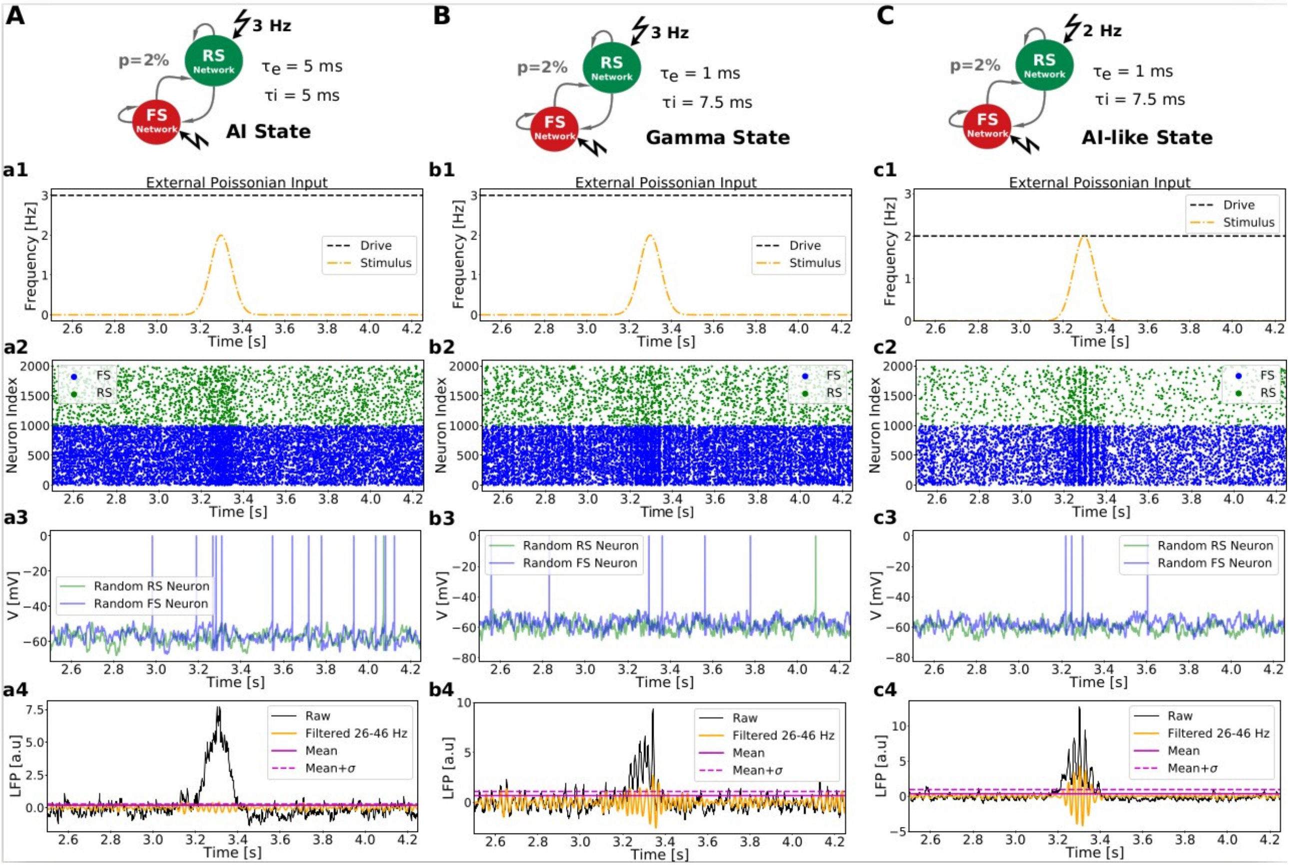

5) *I do not find particularly clear or precise (e.g. it is difficult to distinguish the different shades of green in C right) the depiction of resonance in Fig.9. Plotting the resonance curve amplitude and phase as a function of frequency, as usually done, would seem less colorful but less demanding on the reader's part.*

Answer: We thank the Reviewer for the excellent suggestion, which we followed. We copied the new figure bellow:

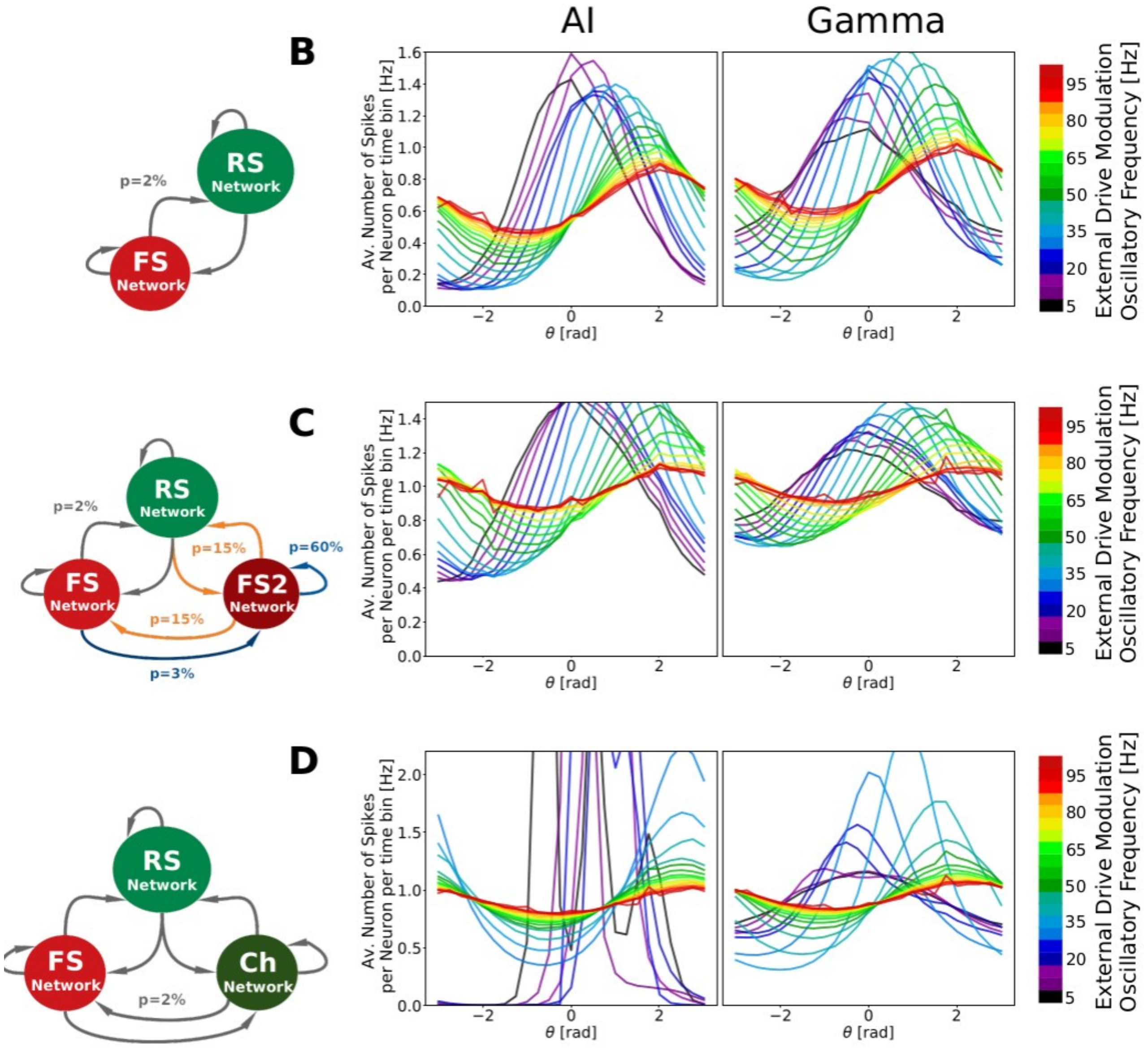

On the other hand, we still believe that the colormap provides a better visualization because one can better see the resonance region in the two dimensions. We changed the color scheme to make it more clear and we included this figure as supplementary.

6) *There are several minor English mistakes that should be corrected ("an stimulus" multiple times, "cells…had its" line 243, "participates of" line 268, odd sentence line 268-269, "an structure" line 299,…)*

Answer: The English corrections have been done.

We thank the Reviewer for very useful and constructive comments.

Reviewer #2:

The authors have analyzed the human electrophysiology data and studied the properties of LFP gamma oscillations and the spiking activity of the single cells. They have shown that only a small percentage of the recorded neurons are phase-locked to the gamma oscillations and show elevated activity during the gamma bursts. They have then developed three different spiking neuronal models that could produce asynchronous activity as well as gamma oscillations through different mechanisms and have explored in what extent they can produce the same properties observed in experimental data. They have also studied the response of the three networks to different types of external stimuli and have concluded that the in all the three networks, larger response is observed when they are in irregular-asynchronous state.

I think the manuscript provides interesting results and can be considered as an important contribution in the context of the dynamics of cortical networks. I just have several comments that might be useful to increase the readability and coherency of the manuscript.

General: *Do the authors implicitly assumed that the gamma bursts emerge through mean input augmentation in Human subject, like the model? This could not always be the case since even with homogeneous input rate in a certain range, the gamma oscillations could spontaneously wax and wane and show quite variable amplitude like what is seen in Ref [82] of the manuscript. I think the authors need to elaborate this point.*

Answer: This is an important point. Our networks, the same way as in the reference [82], are able to generate spontaneous Gamma bursts. On the other hand, these Gamma bursts, both in [82] and in our work, have a very irregular periods of occurrence, which make them impossible to be predicted. Since the spontaneous occurrence of Gamma bursts in this type of model emerges due to fluctuations on the recurrent drive generated by the network dynamics, we decided to regulate this “randomness” by means of a controlled increase of the external input. This choice was merely a practical choice for our protocols of implementation. We did not assume that gamma bursts emerge through mean input augmentation in Human subjects. We added a sentence in the article to clarify this point.

General: *the results show that responsiveness of the simulated networks to external inputs, either pulse packets or rate modulation, is higher at AI state compared with the oscillatory state. However these results could not be readily used to argue in favor of the superiority of the AI state. That is because although the irregular dynamics is advantageous in responsiveness, the signals and information are better transmitted in oscillatory networks (Akam and Kullmann, Neuron 2010; Sherfey et al., Neurobiology of Learning and Memory 2020). I think presence of such a compromise between responsiveness and transmission should be discussed somewhere in the manuscript.*

Answer: We fully agree with this comment and have clarified the manuscript about this. We have reworded the end of the discussion in this regard, and cited the reference suggested. We now describe that AI states, because of their high responsiveness, are well suited to detect inputs, while gamma oscillations, due to their tighter time precision, are better suited to transmit timing information.

Line 14: *“biding”, correct*

Answer: It was corrected

Line 21: *weak pairwise correlations can also be case for the synchronous state when the neurons sparsely fire. Please restate the AI state more precisely.*

Answer: Thank you for this observation. Indeed, the definition was not very well written. We included “stationary global activity”, like it is defined in Amit and Brunel (1997b).

Lines 30 and 31: *I think it is adequate to bring references here; although later in the results the references are introduced.*

Answer: The references were added.

Lines 70 to 76: *with this arrangement it seems that the inputs to the neurons have a low degree of correlation that certainly affects the dynamics and make it different with the case where the inputs are completely uncorrelated. Can the authors quantify this correlation or at least indicate the presence of such correlation?*

Answer: You are right, the way we implemented the network drive inserts some correlation among neurons. We adopted this drive method for technical reasons. In *Brian2, the* simplest way to implement a time varying drive is by using a function that requires this correlation (PoissonGroup). We also verified our results with simulations in which each neuron received different and independent spikes trains with no time dependence (this is implemented in *Brian2* by means of the function *PoissonInput*). With this methods our results were qualitatively the same. A sentence was added to the article to indicate the presence of such correlation.

Line 81: *for me the decay time of the excitatory connections are smaller than those usually used in other references. Can the authors justify their choice?*

Answer: For PING network, we made a parameter search (with respect to the synaptic decays) in such a way that the oscillations in the Gamma band would be present in the network composed of neurons with neuronal parameters chosen in accordance with previous publications of our group (Zerlaut & Destexhe – Neuron 2017, Zerlaut et al. - Journal of Computational Neuroscience 2018). We added a Supplementary Figure (S1, copied bellow) showing the parameter search done to arrive to this choice. See its legend in the edited article.

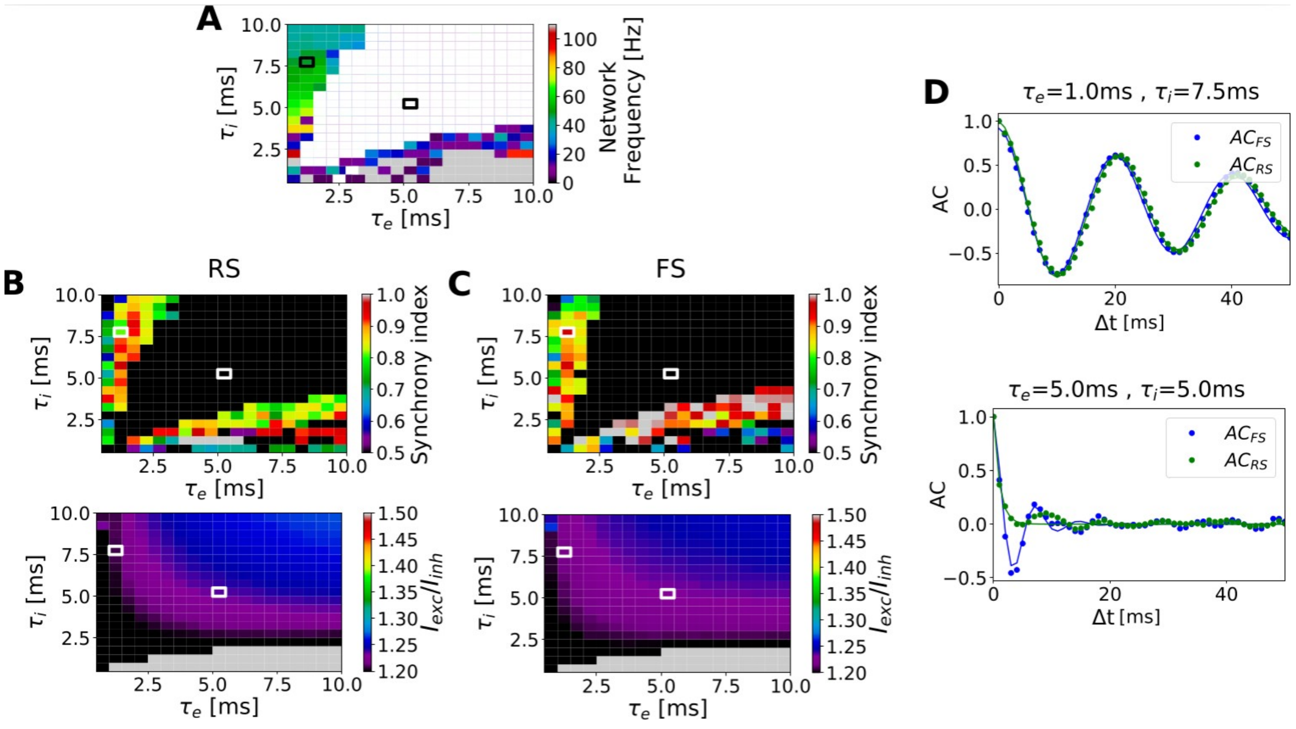

Line 81: *or-->and ?*

Answer: We exchanged “and” by “(“

Line 83: *“were computed inside of the synaptic current term”. I think this can be reworded.*

Answer: This sentence was reformulated in the general paragraph preceding the detailed description of each network.

Lines 94 to 96: *again the choice of the decay times is not justified. Do the authors implicitly mean that for an AI state the synaptic decay times should be equal? A reference or more clarification might help here. General in methods: It seems that subscript for “external” is used with capital initials in some cases and lowercase in others. Please make it coherent.*

Answer: It is true that the way we wrote, we gave the impression that there is no oscillation because the time scales are the same. In fact, this is only a particular case rather than a general rule. We corrected the sentence in the article to make it clear.

These time scales were chosen according to the parameter search shown in the supplementary figure S1, and were also previously used in other publications of our group (Zerlaut & Destexhe – Neuron 2017, Zerlaut et al. - Journal of Computational Neuroscience 2018). As Figure S1D shows, the autocorrelation of the population frequency decays very fast for τe= τi= 5ms , while it is periodically high τe= 1ms , τi= 7.5 ms for other time differences (Δt) .

In addition the subscript for “external” was corrected to have capital initials in all cases.

Lines 144 to 147: *I think more details should be brought for how LFP is calculated.*

Answer: As suggested, we added more details in the methods section.

Lines 148 to 159: *definition of the gamma bursts in the experiments is ambiguous to me.*

1) *First, I see that in many cases gamma oscillations are present outside the gamma burst and since the comparison is made with the baseline average, the outcome depends on if the baseline is itself an oscillatory state or is an asynchronous one.*

Answer: You are right, the outcome do depends on if the baseline is itself an oscillatory state or if it is an asynchronous one.

For the experimental data, we followed the same procedure adopted in the reference (Le Van Quyen et al. - Pnas 2016) which used a deviation of 2 standard deviation from the baseline during at least 3 Gamma cycles. This criteria were not enough to identify all Gamma bursts (some Gamma bursts were ignored), on the other hand, all identified Gamma bursts were in fact Gamma bursts (no false positives were included in the analysis). All the Gamma bursts automatically identified by the algorithm were individually confirmed visually.

For the data coming from our neural networks, the Gamma bursts were generated due to the increase of external drive (like is show in Fig 4). The duration of the drive increase was: 500 ms (plateau) plus 50 ms of linear increase in the beginning and 50 ms of linear decrease in the end. The inter-burst duration of 3 seconds. Not all periods of increased drive generated Gamma bursts. We used the same algorithm applied to the experimental data, but instead of using 2 standard deviations from the mean of Hilbert envelope, we used 1 standard deviation. Again, with this criteria choice, some Gamma bursts were ignored but no false positives were included in the analysis. In addition, as we did in the experimental data, all identified Gamma bursts were individually confirmed visually.

A sentence was added to the article in Methods section to clarify this point.

2) *Moreover, in experimental study with multiple electrodes (referring to Fig 1A), it seems that the gamma burst can be observed in different times for different electrodes, or simultaneously for all the electrodes. This latter leads to the question that which is considered in the calculation of ~13 ms of the total time of gamma bursts in each recording session. I hope that the authors can make this point more clear.*

Answer: The identification of Gamma bursts was done separately for each electrode. This way, neurons measured in particular electrode, had their phases and firing rates analyzed exclusively with respect to the rhythm measured in this electrode. The Gamma duration per data segment, indicated in Figure 2D, is the average Gamma duration among all the electrodes in that segment. The 13 ms is the average of this averages (as indicated in the figure).

We added some sentences both in Figure 2 and in the methods sections to clarify this point.

3) *For example, one can think that if the criterion for the gamma bursts is moderated, how the results change? Or if the criterion is chosen an absolute one instead of a differential one?*

Answer: Since no false positive was included in the analysis, we believe this result is robust.

Line 154: *could a reference be added for Keiser filter?*

Answer: In fact, this was a typo. It should have been written Kaiser, not Keiser. We added more details about the filter and the references.

L*ines 169 and 170:* *check if the capital are needed.*

Answer: No, it is not needed. This was corrected.

Lines 239 to 241: *how this result can be inferred from the figures?*

Answer: Thank you for this observation. This result was not explicitly shown. We changed the paragraph order and added the following supplementary figure (S6) in which this result is better explained (see figure legend in the article).

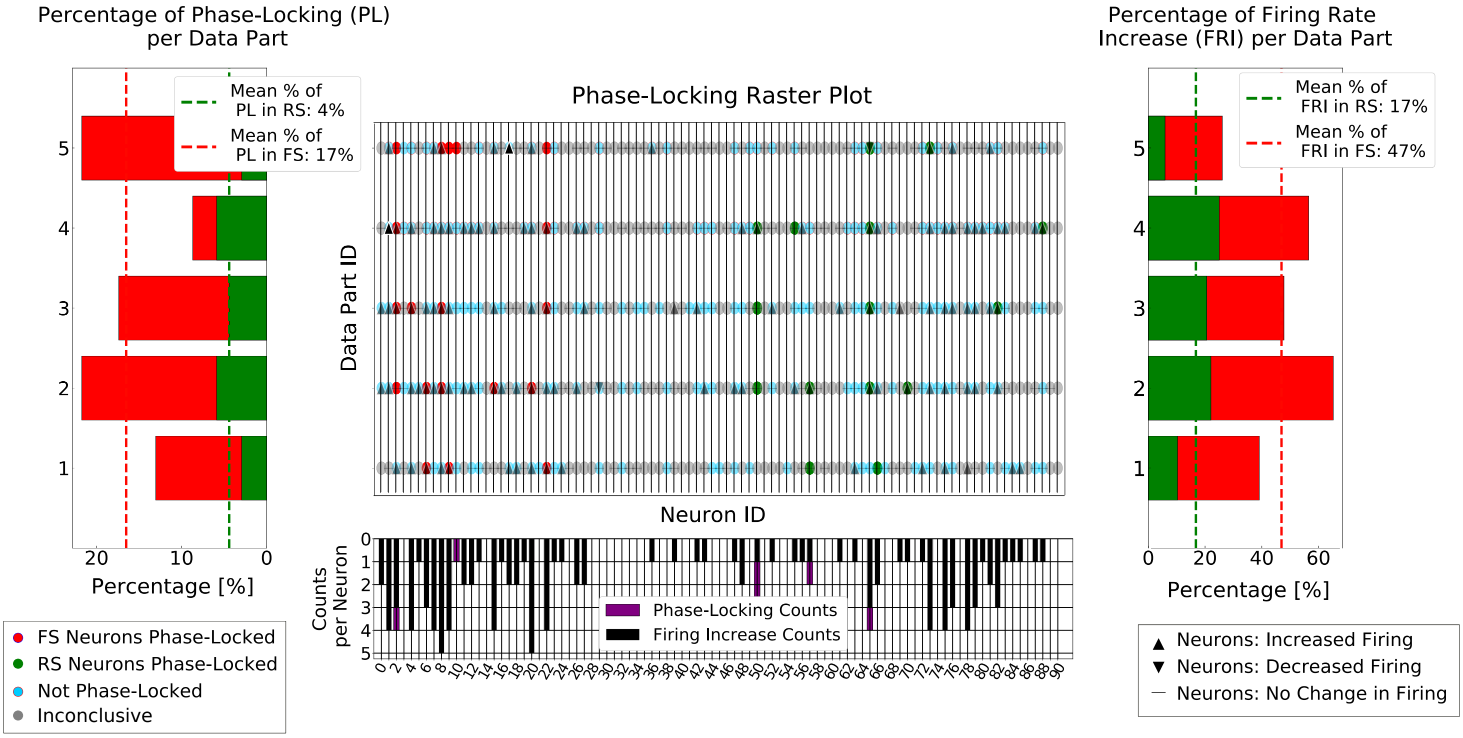

Line 248: *I think that a major un-addressed point is that if the neurons that show higher rate in the gamma bursts are those that show higher phase locking? Could this analysis be made or could it be discussed?*

Answer: To address this point we provided a supplementary figure (S9), attached bellow. This figure depicts the average firing rate of each neuron in each of the 5 data segments as a points (each neuron presents 5 points). The color of each point corresponds to the neuron classification with respect to phase-locking in the correspondent data segment (purple: phase-locked, red: not phase-locked and gray: inconclusive). More details are given of the figure legend.

Figure S9 indicates that there is no simple correlation between firing rate inside Gamma bursts and the fact of being phase-locked. In this, we see cells with high firing rates not being phase-locked, the same way as cells with lower firing rates being phase-locked. The human data set used is too small to be able to arrive to any conclusion. The same is true if we try do drive conclusions about the co-occurrences of firing rate increase and phase-locking (see Figure S6)

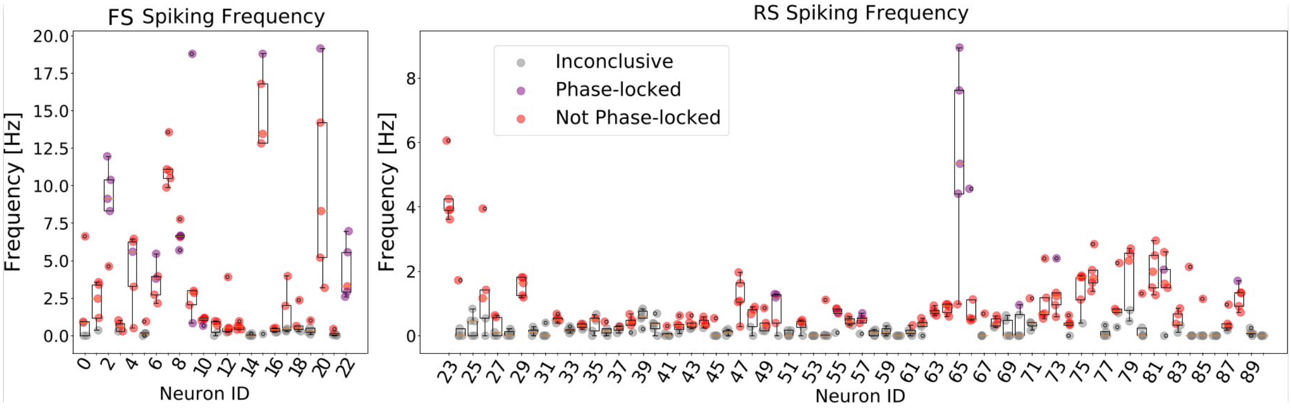

Fig 2: *please indicate the shaded region in panel B and C are, presumably, the burst period. Moreover, I suggest to increase the size of panel A or only show number of sample electrodes for more clarity.*

Answer: We indicated the burst period as a shaded region.

Fig 4 and general: *some legends of the figures are too small and cannot be read when the size of paper is standard. Please recheck all the figures.*

Answer: We adjusted legend, ticks and axe label sizes in all main figures.

Line 345: *"Fig 5 illustrates how network heterogeneities in network connections (ING Network) or in neuron types (CHING Network) influence network activity." I understand what the authors meant but the sentence gives a sense that the effect of heterogeneity is systematically studied in the figure. Please moderate the expression.*

Answer: We agree with the reviewer, we used the word "heterogeneity" in an ambiguous fashion. What we actually meant was “variations of connectivity”. We rephrased the sentence to “*Fig 5* illustrates how different network stuctures (different connectivities in the ING Network or different neuron types in the CHING Network) influence network activity”

Line 370: *weaker than?*

Answer: With this observation it is not clear if the reviewer is talking about the typo or the fact that I_{AMPA}/I_{GABA} ratio in excitatory cells is weaker than in inhibitory cells. If the question is about the fact of being weaker, this can be seen in the parameter search done in the new supplementary figure (S1B and S1C) and is explained in detail in references: Brunel & Wang - J. Neurophysiology 2003 and Geisler et al. - J. Neurophysiology 2005.

Line 418: *“a restrict group” please correct.* **Answer:** This was corrected.

Line 723: *“It had an amplitude of 50Hz” could be reworded, and meddle->middle?*

Answer: The sentence was reworded an all occurrences of the misspelling were corrected.

We thank the Reviewer for very useful and constructive comments.
